# ArpC5 isoforms regulate Arp2/3 complex–dependent protrusion through differential Ena/VASP positioning

**DOI:** 10.1126/sciadv.add6495

**Published:** 2023-01-20

**Authors:** Florian Fäßler, Manjunath G. Javoor, Julia Datler, Hermann Döring, Florian W. Hofer, Georgi Dimchev, Victor-Valentin Hodirnau, Jan Faix, Klemens Rottner, Florian K.M. Schur

**Affiliations:** ^1^ Institute of Science and Technology Austria (ISTA), Klosterneuburg, Austria.; ^2^ Zoological Institute, Technische Universität Braunschweig, Braunschweig, Germany.; ^3^ Department of Cell Biology, Helmholtz Centre for Infection Research (HZI), Braunschweig, Germany.; ^4^ Institute for Biophysical Chemistry, Hannover Medical School, Hannover, Germany.

## Abstract

Regulation of the Arp2/3 complex is required for productive nucleation of branched actin networks. An emerging aspect of regulation is the incorporation of subunit isoforms into the Arp2/3 complex. Specifically, both ArpC5 subunit isoforms, ArpC5 and ArpC5L, have been reported to fine-tune nucleation activity and branch junction stability. We have combined reverse genetics and cellular structural biology to describe how ArpC5 and ArpC5L differentially affect cell migration. Both define the structural stability of ArpC1 in branch junctions and, in turn, by determining protrusion characteristics, affect protein dynamics and actin network ultrastructure. ArpC5 isoforms also affect the positioning of members of the Ena/Vasodilator-stimulated phosphoprotein (VASP) family of actin filament elongators, which mediate ArpC5 isoform–specific effects on the actin assembly level. Our results suggest that ArpC5 and Ena/VASP proteins are part of a signaling pathway enhancing cell migration.

## Teaser

Arp2/3 complex isoform specificity defines branch junction structures, actin network geometry and cell migration.

## INTRODUCTION

The actin filament nucleating the Arp2/3 complex is involved in essential cellular processes such as endocytosis, cell shape control, DNA repair, and cell migration (*1*–*3*). The Arp2/3 complex also plays important roles in pathological conditions, such as in the actin cytoskeleton–dependent motility of selected viruses and bacteria or cancer metastasis (*4*–*7*).

**Fig. 1.**
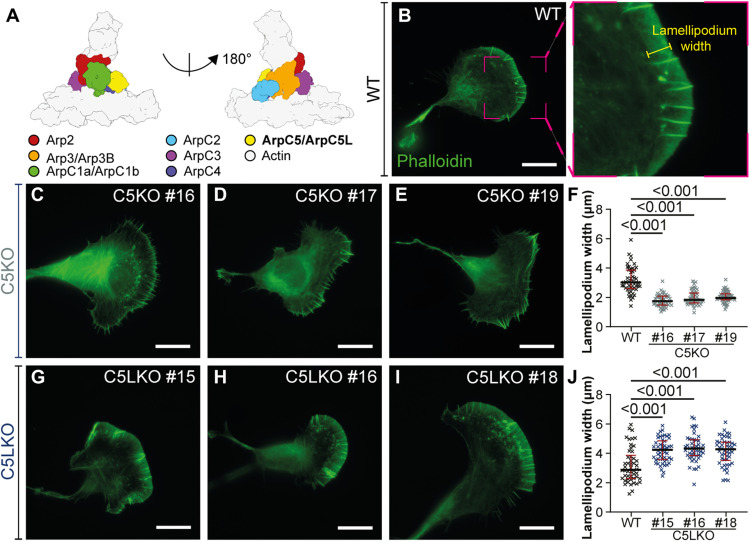
ArpC5 isoforms affect lamellipodium morphology. (**A**) Schematic representation of the branch junction highlighting the positions of the individual Arp2/3 complex subunits within this assembly. Subunit colors are annotated. (**B** to **J**) Analysis of lamellipodium width phenotype. (B to E and G to I) Representative epifluorescence micrographs of B16-F1 wild-type (WT) (B), C5KO (C to E), and C5LKO (G to I) cells visualizing the actin cytoskeleton using fluorescent phalloidin. For the knockout (KO) cells, three individual clones are shown. The right panel in (B) shows a zoom-in of the indicated area in the left panel and exemplifies which kind of areas were considered as lamellipodia for width measurements. Three such measurements were performed per cell, and their average was then used for statistical analysis. (F) Lamellipodium width of B16-F1 C5KO lines and B16-F1 WT cells. Kruskal-Wallis test combined with Dunn’s multiple comparison test, *n* = 50 cells for each experimental group, *P* values shown in the chart. Black lines indicate medians (3.020, 1.742, 1.830, and 1.972 μm), and red lines indicate quartile ranges. (J) Lamellipodium width of B16-F1 C5LKO lines and B16-F1 WT cells. Kruskal-Wallis test combined with Dunn’s multiple comparison test, *n* = 50 cells for each experimental group, *P* values shown in the chart. Black lines indicate medians (2.884, 4.237, 4.324, and 4.280 μm), and red lines indicate quartile ranges. Scale bars, 20 μm.

The Arp2/3 complex, consisting of the two actin-related proteins (Arp) 2 and Arp3 and five scaffolding subunits ArpC1 to ArpC5 (Fig. 1A), generates branched actin networks via nucleating daughter actin filaments on the sides of preexisting mother filaments (*2*, *8*–*11*). This leads to the formation of so-called actin filament Arp2/3 complex branch junctions showing a defined geometry of ~70° (*2*, *10*, *12*–*15*). As the Arp2/3 complex is able to nucleate its own substrate, branched actin networks are potentially capable of exponential growth. This requires Arp2/3 complex activity to be tightly regulated to determine actin network turnover and stability, with regulation occurring both upstream and downstream of Arp2/3 complex–mediated branch junction formation (*5*). Specifically, Arp2/3 complex activation is driven by nucleation-promoting factors (NPFs), such as members of the Wiskott-Aldrich syndrome protein family. In addition, Cortactin binds to branch junctions to increase their stability and might enhance the release of NPFs (*16*).

The Arp2/3 complex is not a uniform macromolecular entity but can exist in eight different subunit compositions, caused by the presence of Arp3a/Arp3B, ArpC1a/ArpC1b, and ArpC5/ArpC5L subunit variants (commonly referred to as isoforms in the literature). This compositional heterogeneity of the Arp2/3 complex allows for fine-tuned nucleation activity and branch junction stability (*17*–*19*). Certain ArpC1-ArpC5-isoform combinations seem to be preferentially assembled into one complex (*17*). Together, these mechanisms define the overall activity of the complex in in vitro reconstituted actin networks or in vaccinia virus (VACV) actin tails in infected cells, with ArpC1a and ArpC5 being associated with lower Arp2/3 activity compared to the combination of ArpC1b and ArpC5L (*20*). Specifically, Arp2/3 complex isoform–specific activity was described to depend on the preferred stabilization of ArpC5L-containing branches by Cortactin against Coronin-induced debranching (*17*). However, it can be anticipated that additional regulatory layers beyond Cortactin stabilization of isoform-specific branch junctions are at play to modulate Arp2/3 complex activity.

Single-particle cryo–electron microscopy (cryo-EM) of ArpC1a/ArpC5 and ArpC1b/ArpC5L-containing recombinant Arp2/3 complexes showed slightly different stability of the ArpC5 isoforms in the inactive complex but did not otherwise reveal substantial structural differences in the different Arp2/3 complex variants. This suggests either that their specific activities result from different energetic barriers for activation (*2*) or that major structural differences, which cause changes in nucleation activity, are only prevalent in the active conformation of the Arp2/3 complex within the branch junction. These exciting findings warrant further studies to identify isoform-specific Arp2/3 complex interactions with proteins upstream or downstream of branch junction nucleation.

Moreover, a better understanding of how isoform-specific properties of the Arp2/3 complex might differ among biological systems is required. Intriguingly, the ArpC5L isoform has been shown to increase Arp2/3 complex activity (*17*), a trait normally associated with enhanced cell migration and metastasis (*21*–*24*). In contrast, elevated ArpC5 levels have been linked to cancer progression (*25*–*27*), as ArpC5 depletion has reduced the motility and invasiveness of carcinoma cells (*25*). This raises the question whether and how ArpC5/5L isoforms uniquely affect actin networks involved in cell migration.

An established model to study the regulation of branched actin networks in cell motility is the lamellipodium, a thin sheet-like protrusion at the leading edge of migrating cells densely filled with branch junctions (*28*–*30*). The prototypical initiation of lamellipodia depends on the activation of the Arp2/3 complex via the NPF *Wiskott-Aldrich syndrome protein family verprolin-homologous protein* (WAVE) regulatory complex (WRC). Polymerization at free barbed ends of Arp2/3 complex–nucleated filaments is thought to be enhanced by the activity of actin filament elongating proteins of the Ena/Vasodilator-stimulated phosphoprotein (VASP) family (*31*–*33*). However, it should also be noted that Ena/VASP proteins can potentially counteract lamellipodial Arp2/3 complex accumulation, as implied by genetic Ena/VASP disruption (*32*). Because of their morphological simplicity and their accessibility to a large variety of experimental approaches, lamellipodia have been used as model systems across different disciplines, from cell biology to structural biology. Genetic engineering of migratory cell lines via CRISPR-Cas9 has been successfully used to delineate lamellipodium formation and to understand the activity of several important actin-binding proteins (ABPs), such as WAVE and its isoforms (*34*), lamellipodin (*35*) or formin-like (FMNL) subfamily formins (*36*), and the capping protein regulator Twinfilin (*37*). The lamellipodium has also been used extensively to study the ultrastructure and topology of actin networks under varying experimental conditions using EM approaches (*15*, *28*, *29*, *32*, *38*–*41*). We have recently been able to exploit the high number of branch junctions in lamellipodia in a cellular structural biology approach using cryo–electron tomography (cryo-ET) to determine the structure of the actin filament Arp2/3 complex branch junction to subnanometer resolution (*13*). This allowed for a more precise understanding of the structural transitions that occur upon Arp2/3 complex activation and interactions that are formed with actin mother and daughter filament. The structural description of the mammalian branch junction has been recently extended to a 3.9-Å-resolution single-particle cryo-EM structure of in vitro reconstituted actin filament Arp2/3 complex branch junctions (*12*). However, as the branches observed in our previous in situ study or in the aforementioned in vitro work both contained all possible Arp2/3 complex isoform compositions, no insights into isoform-specific branch junction structures could be obtained.

To explore ArpC5 isoform–dependent actin network organization and related cell migration characteristics, we chose a multiscale approach combining recent developments in the genetic engineering of cells and cellular structural biology. This offered the opportunity to integrate high-resolution information on isoform-specific branch junction structures and the resulting ultrastructural cytoskeleton architectures with Arp2/3 complex isoform–specific cellular morphology, actin network dynamics, and ABP localization. To this end, we have generated KO cell lines lacking either one or both of the ArpC5/5L isoforms and describe across multiple experimental scales how the two distinct ArpC5 isoforms differentially regulate branched actin networks in cell migration. We reveal that incorporating a specific ArpC5 isoform into the Arp2/3 complex determines lamellipodial protrusion characteristics, protein dynamics, and actin network ultrastructure. While cells exclusively containing ArpC5L exhibited narrower and less protrusive lamellipodia, ArpC5-containing cells displayed even wider lamellipodia than wild-type (WT) cells. In explaining this phenotype, our data suggest that the Arp2/3 complex not only is a nucleator of actin filaments but also influences actin elongation characteristics in an ArpC5/5L isoform–dependent fashion, e.g., by defining the positioning of actin filament elongation proteins of the Ena/VASP family at the leading edge. Therefore, our results provide novel insights into why specifically ArpC5 over ArpC5L is associated with enhanced cell motility and could be linked to increased cancer progression and metastasis (*25*–*27*).

## RESULTS

### ArpC5 isoform composition determines protrusion morphology

To explore the role of the two ArpC5 isoforms in lamellipodia architecture and cell migration, we used a CRISPR-Cas9 knockout (KO) approach (*42*) to generate B16-F1 mouse melanoma cells lacking either ArpC5 (referred to as C5KO), ArpC5L (C5LKO), or both isoforms (C5/C5LKO). Recent proteomics data on B16-F1 cells revealed a ratio of endogenous ArpC5 to ArpC5L of 3.8 to 1 (*21*). For all generated cell lines, successful KO was verified in three independent clones via genomic sequencing (fig. S1) and confirmed by complete loss of ArpC5 and/or ArpC5L proteins by Western blotting (fig. S2A).

Double KO cell lines lacking both ArpC5 and ArpC5L isoforms were completely devoid of lamellipodia (fig. S3). These results indicate that the actin assembly activity of Arp2/3 complexes lacking an ArpC5 subunit reported in vitro (*43*) is insufficient to maintain branched actin networks in lamellipodia. Notably, single-isoform KO cells revealed opposing lamellipodial phenotypes (Fig. 1, B to J). While C5KO cells exhibited significantly narrower lamellipodia than B16-F1 WT cells (Fig. 1F), the lamellipodial width of C5LKOs was significantly increased (Fig. 1J). In addition, C5KO cells often exhibited a large number of finger-like protrusions (Fig. 1, C to E). To corroborate our observations of these isoform-specific lamellipodia phenotypes, we generated C5KO and C5LKO lines in Rat2 fibroblasts (figs. S1 and S2B). Loss of either isoform in Rat2 cells resulted in lamellipodial phenotypes highly similar to B16-F1 cells (fig. S4), demonstrating that observed effects are consistent across at least two different species and cell types. As all lines of a given KO showed consistent phenotypes, we continued our experiments with one selected individual clone for each condition (for B16-F1 C5KO #17, C5LKO #16, and C5/C5LKO #20, and for Rat2 C5KO #4 and C5LKO #11).

**Fig. 2.**
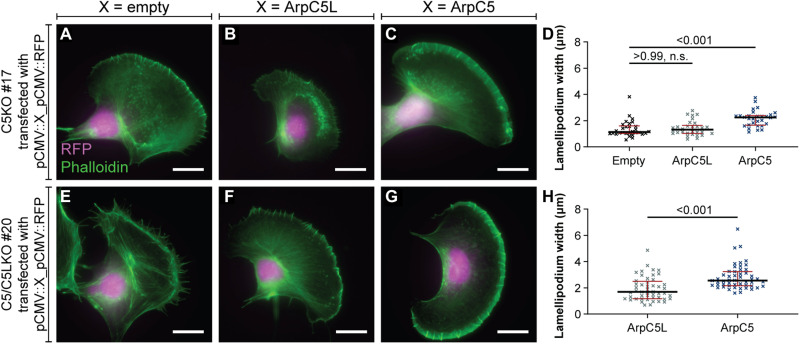
The lamellipodial C5KO phenotype is mediated by isoform specificity and not by gene dose effects. (**A** to **C**) Representative epifluorescence micrographs of B16-F1 C5KO cells transfected with pCMV::empty_pCMV::RFP (A), pCMV::ArpC5L_pCMV::RFP (B), and pCMV::ArpC5_pCMV::RFP (C) visualizing the actin cytoskeleton using fluorescent phalloidin and verifying transfection via RFP fluorescence. (**D**) Quantitation of lamellipodium width of B16-F1 C5KO cells transfected with the same constructs as in (A) to (C). Measurements were done as shown in Fig. 1B. Kruskal-Wallis test combined with Dunn’s multiple comparison test, *n* = 30 cells per experimental condition, *P* values shown in the chart. Black lines indicate medians (1.143, 1.310, and 2.248 μm), and red lines indicate quartile ranges. (**E** to **G**) Representative epifluorescence micrographs of B16-F1 C5/C5LKO cells transfected with pCMV::empty_pCMV::RFP (E), pCMV::ArpC5L_pCMV::RFP (F), and pCMV::ArpC5_pCMV::RFP (G) visualizing the actin cytoskeleton using fluorescent phalloidin and verifying transfection via RFP fluorescence. (**H**) Quantitation of lamellipodium width of B16-F1 C5/C5LKO cells transfected with the same constructs as in (F) and (G). No values are given for cells transfected with pCMV::empty_pCMV::RFP as those did not exhibit lamellipodia. Mann-Whitney test, *n* = 51 and 49 with *P* values shown in the chart. Black lines indicate medians (1.698 and 2.549 μm), and red lines indicate quartile ranges. Scale bars, 20 μm, n.s., not significant.

To test whether the reduced lamellipodium width observed in C5KOs is caused by isoform-specific mechanisms or is the result of a dosage effect (e.g., via reducing the overall ArpC5/ArpC5L pool and thus the number of functional Arp2/3 complexes), we performed rescue experiments in a B16-F1 C5KO and C5/C5LKO background (Fig. 2, A to H). We transiently transfected cells with dual expression plasmids encoding either red fluorescent protein (RFP) alone, RFP and ArpC5L, or RFP and ArpC5 and quantified the lamellipodium width of transfected cells as identified by RFP expression. In this setup, only the construct expressing ArpC5 was able to rescue the lamellipodium phenotype of C5KO cells, resulting in WT-like cells (Fig. 2, C and D). In contrast, transfection with the ArpC5L-encoding construct did not rescue lamellipodia width (Fig. 2, B and D). Repeating the same transfection approach in the C5/C5LKO background (Fig. 2, E to H) confirmed these observations and also the functionality of our ArpC5 and ArpC5L encoding vectors because the expression of ArpC5L or ArpC5 in double KO cells again resulted in the establishment of narrow or wide lamellipodia, respectively (Fig. 2H). To establish that in our rescue experiments the transfection of B16-F1 cells with ArpC5- and ArpC5L-encoding vectors results in the expression of sufficient amounts of the complementing proteins, we analyzed, by quantitative Western blotting, the extent of ArpC5 and ArpC5L overexpression in the C5/C5LKO #20 background. This analysis revealed that protein levels of ArpC5 and ArpC5L are approximately fourfold higher after transfection with the respective vectors compared to B16-F1 WT (fig. S5) and hence are not limiting for forming functional Arp2/3 complexes.

Considering that a reduction of ArpC5 protein levels via knockdown had been shown to decrease the abundance of ArpC1a in HeLa cells (*17*), we also tested whether, in C5KO or C5LKO cells, the levels of other Arp2/3 complex subunits were affected as well, potentially altering overall Arp2/3 complex activity. We assessed the relative abundance of ArpC5, ArpC5L, Arp3, Arp2, ArpC1b, and ArpC1a in B16-F1 and Rat2 single KO lines using a quantitative Western blot approach (fig. S6 and table S1). However, we did not observe a marked change in combined Arp3 and Arp2 abundance (table S1). The only considerable changes consistent across both cell types were a doubling of ArpC5L levels in C5KOs (2.34 in B16-F1 C5KO #17 and 2.16 in Rat2 C5KO #4) and an increase of ArpC1a levels in C5LKOs (1.79 in B16-F1 C5LKO #16 and 1.38 in Rat2 C5LKO #11; table S1). Although we did not assess the number of intact Arp2/3 complexes directly, our data indicate that overall Arp2/3 complex levels should not be altered as compared to the respective WT cell lines, and further underlines that the C5KO phenotype is not caused by a gene-dose effect.

As the results of the rescue experiments were consistent with ArpC5 and ArpC5L having isoform-specific functions in a lamellipodial context, we explored the putative mechanisms mediating the observed differences. Such mechanisms can include altered Arp2/3 complex protein levels (which we already analyzed; fig. S6 and table S1), selective interactions with Arp2/3 complex regulators, up-regulation of competing actin nucleators, ultrastructural alterations in the architecture of branched actin filament networks, structural differences of Arp2/3 complex branch junctions resulting in differences in branch nucleation activity, stabilization, and turnover, or, lastly, differential recruitment of downstream factors.

### Reduced lamellipodium width in C5KO cells is not due to subcellular ArpC5L or actin redistribution

Recruitment of specifically ArpC5L or large quantities of actin to intracellular locations other than the lamellipodium could explain the C5KO lamellipodial phenotype. First, to test for ArpC5 isoform–dependent recruitment of Arp2/3 complexes to different locations within the cell, we performed immunofluorescence assays using antibodies against ArpC5 and ArpC5L (fig. S7). After confirming the isoform specificity of the antibodies using B16-F1 C5KO and C5LKO lines (fig. S7, A to F), we found the distribution of endogenous ArpC5 and ArpC5L between the lamellipodium and the cell body to be highly similar to B16-F1 control cells (fig. S7, G to J).

Second, to exclude the idea that up-regulation of actin polymerization at structures outside the lamellipodium and, hence, an increased competition for available G-actin caused the C5KO lamellipodial phenotype, we quantified the total actin (monomeric and polymerized) and the polymerized actin levels in B16-F1 WT, C5KO, and C5LKO lines. First, using the intensity of fluorescently labeled F-actin, we assessed the abundance of polymerized actin on a single-cell basis (fig. S7K). We then performed quantitative Western blotting to measure the total actin levels within cells (fig. S7L).

F-actin levels and total actin content in C5KO cells were comparable to WT cells, indicating that the reduced lamellipodium width in C5KO cells is not due to a global competition for G-actin. However, the total actin levels in C5LKO cells were slightly lower in comparison to both WT and C5KO cells, while their overall F-actin content was increased, suggesting that a larger fraction of actin in C5LKO cells is incorporated in polymerized actin populations.

We then sought to confirm that the reduced lamellipodium width in C5KO cells was not due to a local competition between Arp2/3 complex–nucleated branched actin and formin-mediated linear actin, most likely prevalent in the more abundant finger-like protrusions found at the leading edge of C5KO cells. The most promising candidates for formins acting in this context are FMNL2 and FMNL3, which have been shown to create protruding forces at the leading edge (*36*). Correspondingly, using immunofluorescence assays, we identified the finger-like protrusions in C5KO cells to be associated with FMNL2 and 3 (fig. S8, A to F). As FMNL2/3 were, however, not up-regulated but rather down-regulated in both the C5KO and C5LKO cells (fig. S8G and table S1), we conclude that the large number of finger-like protrusions in C5KO cells is the consequence, and not cause, of less productive actin filament branching within the lamellipodium.

### WAVE1 and WAVE2 do not activate the Arp2/3 complex in an ArpC5 isoform–specific manner

Next, we examined whether upstream regulators of the Arp2/3 complex are causative for the observed phenotypes in C5KO and C5LKO cells. The WRC is a central NPF crucial for Arp2/3 complex activation in canonical lamellipodia (*44*, *45*). Using recently published B16-F1 WAVE1 and WAVE2 KO cell lines (*34*), we aimed to test whether the two WAVE isoforms expressed in B16-F1 cells exhibit preferred activation of either ArpC5 or ArpC5L containing Arp2/3 complexes. We again used isoform-specific antibodies against ArpC5 and ArpC5L (specificity demonstrated in fig. S7, A to F) to detect endogenous ArpC5 and ArpC5L in *WAVE1* and *WAVE2* KO cells (fig. S9). ArpC5 and ArpC5L were localized identically at the leading edge in cells lacking either WAVE1 or WAVE2 (fig. S9, D to I). This shows that Arp2/3 complexes with different ArpC5 isoform compositions are not selectively activated by WAVE1- versus WAVE2-containing WRCs. In a WAVE1/2 double KO cell line, neither ArpC5 nor ArpC5L was detected at the cell periphery in standard growth conditions (fig. S9, J to L), in line with the central role of the WRC in establishing Arp2/3-dependent protrusions.

### Cortactin activity does not affect lamellipodia dimension in an ArpC5 isoform–specific fashion

Having excluded differential Arp2/3 complex activation by the WRC as a central contributor to ArpC5 isoform–specific function in lamellipodia, we focused on the well-established branch junction stabilizer Cortactin (*46*, *47*). Cortactin has been described to protect ArpC5L-containing branch junctions from Coronin-mediated debranching (*17*).

We performed Cortactin knockdowns in B16-F1 WT, C5KO, and C5LKO cells (fig. S10). We confirmed the reduction in Cortactin levels at the single-cell level by immunostaining (fig. S10, B, D, F, H, J, and L), as well as at the population level by Western blotting (fig. S10M). We then evaluated changes in lamellipodia phenotypes via phalloidin staining (fig. S10, A, C, E, G, I, and K). Cortactin knockdown did not result in notable differences in lamellipodia dimensions in WT or ArpC5 and ArpC5L KO cells, reminiscent of previous observations in murine Cortactin KO fibroblasts (*48*), but in contrast to observed changes in VACV tail length in HeLa cells depleted of specific ArpC5 isoforms (*17*). We conclude that the changes in lamellipodial dimensions observed upon ArpC5 isoform KOs are not mediated by differential Cortactin activities.

### ArpC5 isoforms affect VACV actin tail length in a cell type–specific manner

Abella *et al.* (*17*) reported VACV actin tails to be shorter after the knockdown of ArpC5L and longer after the knockdown of ArpC5, respectively, representing an inverse phenotype compared to our observations on lamellipodia width in B16-F1 (Fig. 1, C to J) and Rat2 (fig. S4, B to G) KO cell lines.

To determine whether the differential effects on VACV actin tails and lamellipodia after the loss of ArpC5 isoforms represent different regulatory mechanisms between these two experimental systems, we performed VACV tail length measurements in our B16-F1 and Rat2 WT and KO cell lines (fig. S11). In infected B16-F1 cells, VACV tail lengths were reduced in both C5KO and C5LKO cells compared to WT (fig. S11, A to J). However, the tails observed in C5LKO cells were longer than those in C5KO cells. Moreover, in Rat2 fibroblasts, we also observed longer tails in C5LKO cells and shorter tails in C5KO cells compared to WT (fig. S11, K to T). Hence, our observations for VACV tail lengths in an ArpC5/5L isoform–specific background are consistent with our lamellipodial phenotypes, although to variable extents when comparing Rat2 versus B16-F1 cells.

Considering our results and existing literature, ArpC5 isoforms clearly affect VACV actin tail formation but might do so in a cell type–specific manner. We interpreted this as a direct hint toward additional factors being involved in contributing to the outcome of ArpC5 isoform–specific Arp2/3 complex activities not previously anticipated.

### ArpC5 isoforms differentially affect actin network architecture and branch junction structure

**Fig. 3.**
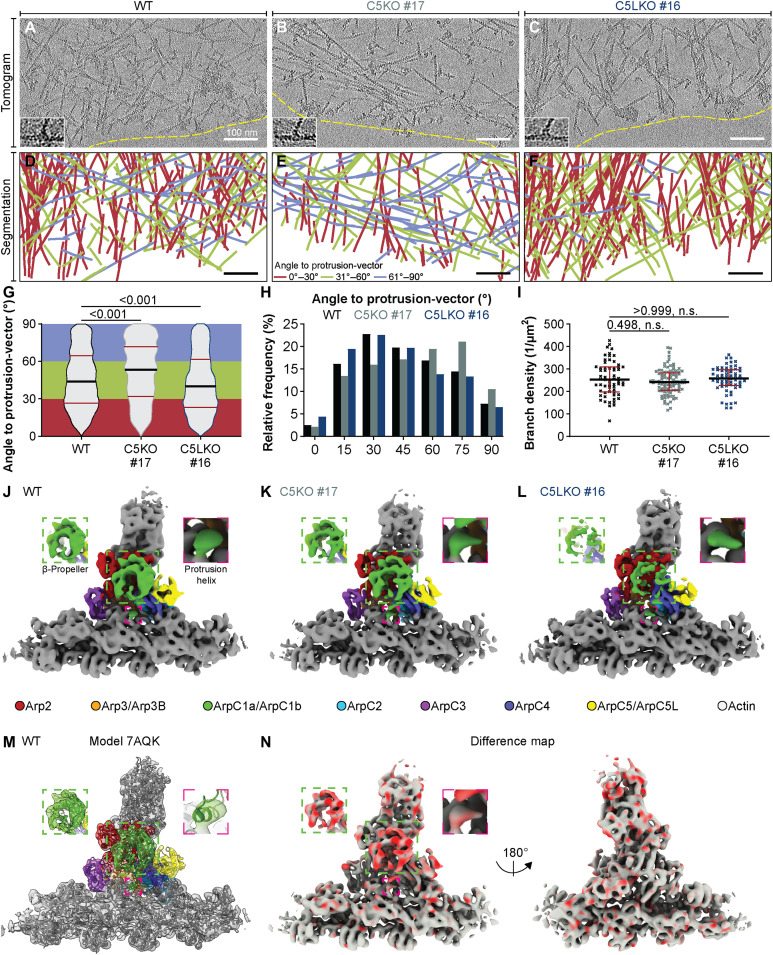
ArpC5 isoforms govern lamellipodial actin network architecture and have differential effects on ArpC1 stabilization in the branch junction. (**A** to **C**) Sums of seven computational slices (~9.5 nm thickness) through cryo-electron tomograms of B16-F1 WT, C5KO, and C5LKO lamellipodia. Dashed yellow lines indicate the leading edge. Insets show enlarged views of representative branch junctions. (**D** to **F**) Segmentations of actin filaments contained in the full height of tomograms shown in (A) to (C). Color coding is given in (E). (**G** and **H**) Visualization of actin filament angular distribution with respect to the vector of protrusion, pooled from 13 tomograms of B16-F1 WT, C5KO, and C5LKO cells each. (G) Violin plot. (H) Histogram. Kruskal-Wallis test combined with Dunn’s multiple comparison test, *n* = 2819, 2195, and 2410 filaments, *P* values shown in the chart. Black lines indicate medians (43.93°, 53.27°, and 40.20°), and red lines indicate quartile ranges. (**I**) Branch density per tomogram in B16-F1 WT, C5KO, and C5LKO lamellipodia. Kruskal-Wallis test combined with Dunn’s multiple comparison test, *n* = 61, 97, and 61 tomograms, *P* values shown in the chart. Black lines indicate medians (252.9, 242.0, and 258.7 μm^−2^), and red lines indicate quartile ranges. (**J** to **L**) Isosurface representations of the actin filament–Arp2/3 complex branch junction derived from B16-F1 WT, C5KO, and C5LKO cells, low-pass filtered to 8.1 Å resolution. Isosurface coloring of Arp2/3 complex subunits is annotated in the figure. (**M**) The EM density map from B16-F1 WT cells (shown transparent) with the rigid body fitted model of the branch junction (PDB 7AQK). Color code as in (J) to (L). (**N**) Difference map (both side views shown) was calculated from the structures of C5KO and C5LKO branch junctions projected onto the map of the C5KO branch junction. Red coloring indicates differences in occupancy. Insets show the ArpC1 subunit β-propeller (green dashed box) and protrusion helix (pink dashed box). Scale bars, 100 nm.

Aiming to understand the differences in the architecture of actin filament networks exhibited by C5KO and C5LKO cells, we used our published cryo-ET specimen preparation protocols (*13*, *49*) to acquire tilt series of vitreously frozen lamellipodia in B16-F1 WT, C5KO, and C5LKO cells (Fig. 3, A to C). We then performed a quantitative ultrastructural analysis of actin network architectures in all cell types (Fig. 3, D to H) using a recently published workflow (*50*). This analysis revealed clear differences in angular distributions of actin filaments between WT and isoform-specific KO lamellipodia (Fig. 3, G and H) and provided an ultrastructural explanation for the respective lamellipodial phenotypes. In C5KO cells, a larger fraction of filaments was oriented parallel to the leading edge. Such filaments do not contribute to the formation of a prominent network with high width (*39*) but rather promote the narrow appearance of the lamellipodium typical for this genotype (Fig. 1, C to F). Correspondingly, in ArpC5L KO cells, a larger number of filaments were found to be oriented almost perpendicular to the leading edge (Fig. 3, F and H), leading to a wider lamellipodium as observed using fluorescence microscopy (Fig. 1, G to J).

One possible explanation for the altered network architectures could be a different nucleation activity of isoform-specific Arp2/3 complexes, resulting in different numbers of branch junctions. To evaluate this possibility, we exploited the high-resolution molecular information present within our cryo-ET data and used classification-corrected template matching (see Materials and Methods) to identify branch junctions within our cryo-electron tomograms. We calculated branch junction densities per square micrometer in lamellipodia of each cell type and found the numbers of branch junctions with medians of 252.9, 242, and 258.7 μm^−2^ for WT, C5KO, and C5LKO, respectively, to be comparable across all three genotypes (Fig. 3I). This is also consistent with our observation that relative abundances of Arp2/3 complex subunits are not strongly altered upon the removal of either ArpC5 or ArpC5L (fig. S6 and table S1).

As our results suggested that overall branch junction formation in lamellipodia is not severely affected by the selective incorporation of either ArpC5 or ArpC5L, we hypothesized that the observed differences in Arp2/3 complex activity might be caused by structural differences at the level of individual branch junctions. To test this, we performed subtomogram averaging and multiparticle refinement on identified branch junctions, yielding 8.0-, 8.1-, and 7.8-Å-resolution structures of actin filament Arp2/3 complex branch junctions from B16-F1 WT, C5KO, and C5LKO cells, respectively (Fig. 3, J to L, and fig. S12). All three structures revealed an overall identical branch geometry of ~70° and corresponded to recently published models of the Arp2/3 complex branch junction (Fig. 3, J to M, and movie S1) (*12*, *13*). Although the overall branch junction was unaltered, we observed a notable difference in the structure derived from C5LKO cells. In this structure, the density of the ArpC1 β-propeller was considerably weaker (Fig. 3L), an observation further confirmed by calculating a difference map between branch junction structures derived from C5KO and C5LKO cells (Fig. 3N). In contrast, the density for the so-called ArpC1 protrusion helix, which tethers ArpC1 to the actin mother filament (*12*, *13*), displayed an equally strong density among all three structures (Fig. 3, J to L and N). These observations suggest that the lack of ArpC1 density in the C5LKO structure is not due to reduced occupancy but could be due to increased flexibility in its β-propeller region with respect to the rest of the branch junction. At their reported resolutions, our cryo-ET densities do not allow us to unambiguously explain how ArpC5 isoforms might affect ArpC1 stability or flexibility. Hence, to obtain a better understanding of how the differences between ArpC5 and ArpC5L, on the primary amino acid sequence and protein structure level, might contribute to the observed structural effect on ArpC1, we referred to a recently determined higher-resolution model of the in vitro branch junction (PDB 7TPT) (*12*). This allowed us to map differences in the primary structure of ArpC5 and ArpC5L to their locations within the quaternary structure (fig. S13) and revealed the largest differences in amino acid sequence between the two ArpC5 isoforms to be located in a nonmodeled linker region within the ArpC5/ArpC5L N terminus. This linker region would be positioned to form contacts with ArpC1, and it is, therefore, tempting to speculate that this stretch is most likely the mediator of isoform-specific effects observed in our cryo-ET densities. This is also consistent with the nucleation activity of Arp2/3 complexes containing ArpC5-ArpC5L-chimeras being dominated by the molecular identity of the N-terminal part of the chimeric protein (*19*).

### ArpC5 isoforms define lamellipodial actin network dynamics and protrusion characteristics

**Fig. 4.**
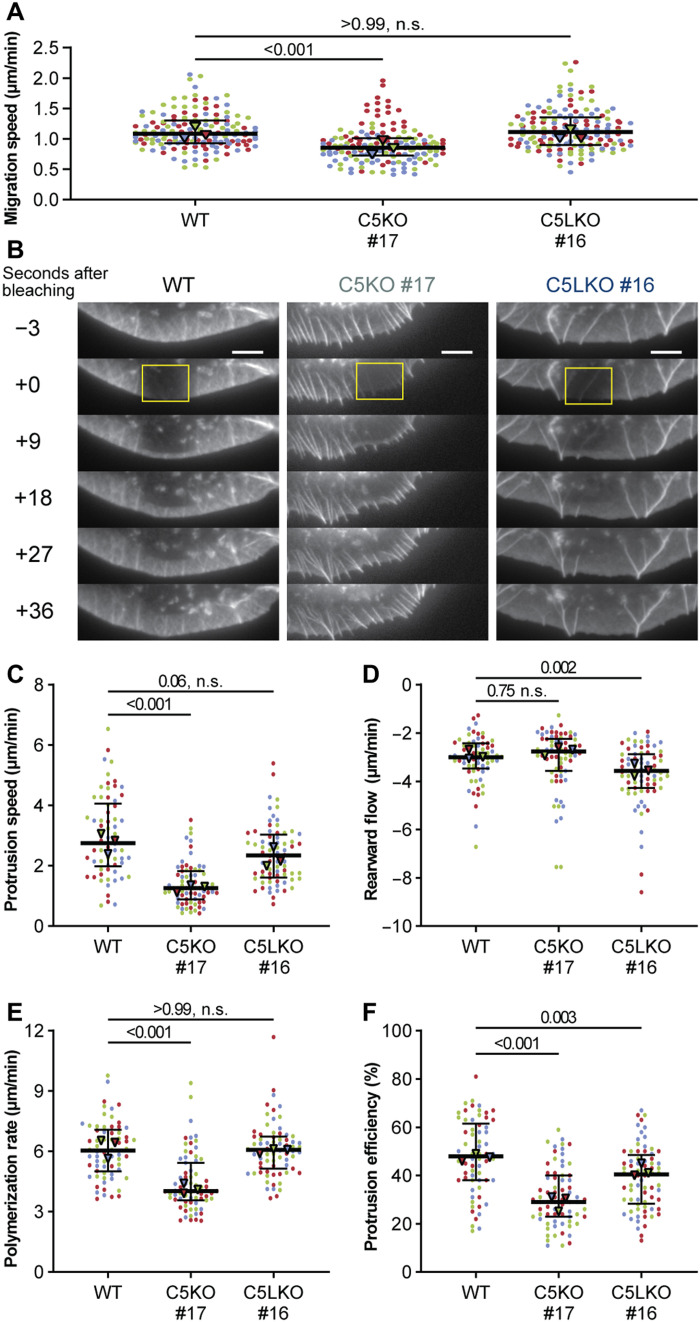
ArpC5 isoforms modulate random migration speed and lamellipodial actin dynamics. (**A**) Random migration speed of B16-F1 WT, C5KO, and C5LKO cells. Kruskal-Wallis test combined with Dunn’s multiple comparison test on pooled data from three independent experiments, *n* = 150 cells per experimental condition, *P* values shown in the chart. Black lines indicate overall medians (1.085, 0.8550, and 1.115 μm/min) and quartile ranges. (**B**) Representative time-lapse images of lamellipodia in B16-F1 WT, C5KO, and C5LKO cells expressing enhanced green fluorescent protein (EGFP)–actin, showing FRAP. Yellow rectangles indicate FRAP regions. (**C** to **F**) Protrusion speed (C), rearward flow (D), polymerization rate (E), and protrusion efficiency (F) as determined via FRAP from B16-F1 WT, C5KO, and C5LKO cells expressing EGFP-actin. Polymerization rate is calculated as the sum of the absolute protrusion speed and the absolute rearward flow. Protrusion efficiency is calculated by dividing protrusion speed by polymerization rate. Kruskal-Wallis test combined with Dunn’s multiple comparison test on pooled data from three independent experiments, *n* = 66, 71, and 72 cells for each experimental condition, respectively, and with *P* values shown in the chart. Black lines indicate overall medians (protrusion speed: 2.747, 1.258, and 2.348 μm/min; rearward flow: −3.003, −2.768, and −3.570 μm/min; polymerization rate: 6.035, 4.026, and 6.068 μm/min; protrusion efficiency: 48.00, 29.00, and 40.50%) and quartile ranges. Data points in (A) and (C) to (F) are color-coded according to the individual experiments. Scale bars, 20 μm. Triangles indicate the medians of the respective experiments.

To explore how ArpC5 and ArpC5L affect actin network dynamics in lamellipodia, we performed live-cell imaging and fluorescence recovery after photobleaching (FRAP) assays (movies S2 to S4). In random migration assays, B16-F1 WT and C5LKO cells exhibited comparable average velocities, while C5KO cells were significantly slower (Fig. 4A). Correspondingly, C5KO cells, while exhibiting comparable retrograde flow to WT, displayed significantly lower protrusion velocity and, thus, lower total actin polymerization rate (Fig. 4, B to E). In contrast, C5LKO cells exhibited a stronger retrograde flow while having comparable overall polymerization rates to WT cells (Fig. 4, B to E; please note that increased rearward flow in panel D is reflected by increased negative values). Although this implied that both KO cell lines exhibited a lower protrusion efficiency than WT (Fig. 4F), the considerably higher polymerization rate found in C5LKO compared to C5KO (Fig. 4E) again clearly marked a difference between the two isoforms and is well in line with the wider lamellipodia observed in C5LKO compared to WT (Fig. 1J). As lamellipodia width in B16-F1 C5LKO cells was increased (Fig. 1, G to J), but actin polymerization rate was comparable to B16-F1 WT (Fig. 4E), we tested for altered depolymerization of F-actin, via quantification of total Cofilin and phospho-Cofilin (p-Cofilin) levels in quantitative Western blot experiments.

Our data show that in both C5KO and C5LKO, inactive Cofilin levels are increased (fig. S14, A and B, and table S1). This increase is more prominent in C5LKO and may, at least in part, contribute to the wider lamellipodia observed in cells of this genotype. In C5KO, however, the reduction of Cofilin activity is difficult to reconcile with the reduced lamellipodium width compared to WT, as it would be expected to rather have the inverse effect.

### Absence of ArpC5 reduces actin filament elongators Mena/VASP at the leading edge

The reduced actin polymerization rate in C5KO cells could be due to either diminished nucleation of filaments or a reduced elongation of existing filaments. As the number of branch junctions is comparable in all cell lines (Fig. 3I) and previous work established that ArpC5L-containing Arp2/3 complexes can mediate more efficient actin assembly and branching (*17*), we excluded the idea that Arp2/3 complex–dependent de novo nucleation of filaments is impaired.

**Fig. 5.**
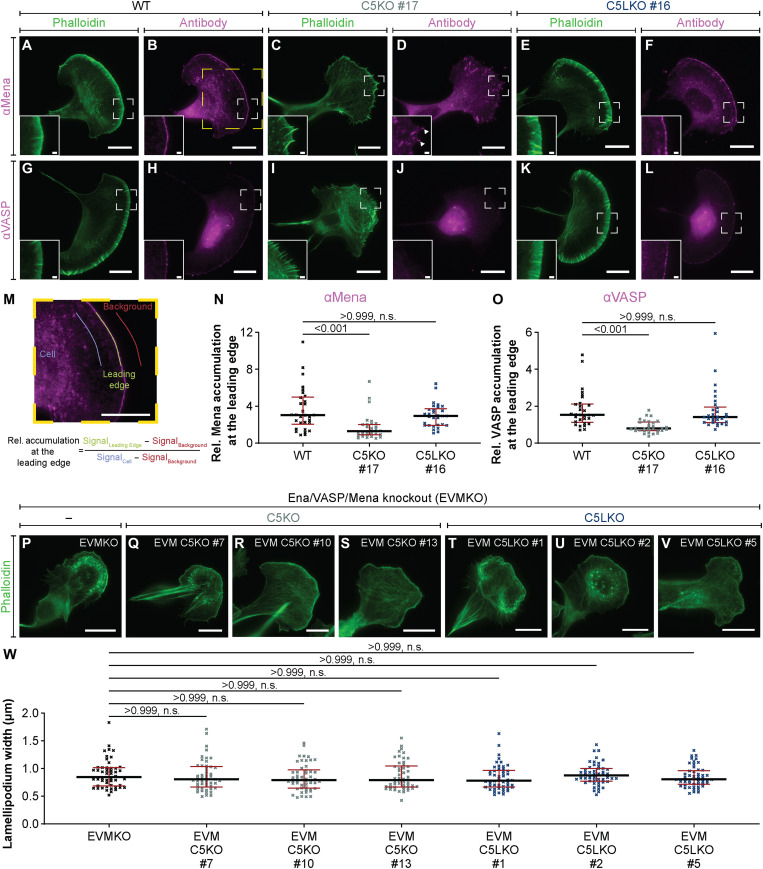
ArpC5 isoforms modulate Mena/VASP recruitment to the leading edge. (**A** to **L**) Representative epifluorescence micrographs of B16-F1 WT (A, B, G, and H), C5KO (C, D, I, and J), and C5LKO (E, F, K, and L) cells visualizing the actin cytoskeleton (A, C, E, G, I, and K) and the localization of Mena (B, D, and F) and VASP (H, J, and L). Insets show magnified areas indicated in respective panels. Arrowheads annotate finger-like protrusions labeled by the anti-Mena antibody. (**M**) Zoom-in on the region indicated in (B), visualizing the measurements and calculations determining the relative accumulation of Mena/VASP at the leading edge. The leading edge was traced for at least 15 μm per measurement. To measure background and cell intensities, the leading edge region of interest was shifted by ~10 μm inside or outside cells while avoiding extremely bright secondary antibody aggregates. (**N** and **O**) Quantitative analysis of the relative Mena (N)/VASP (O) accumulation at the leading edges of B16-F1 WT, C5KO, and C5LKO cells. Kruskal-Wallis test combined with Dunn’s multiple comparison test, *n* = 30 cells for each experimental condition, for both Mena and VASP, *P* values shown in the chart. Black lines indicate medians [Mena (N): 3.044, 1.292, and 2.954; VASP (O): 1.533, 0.8091, and 1.419], and red lines indicate quartile ranges. (**P** to **V**) Representative epifluorescence micrographs of B16-F1 Ena/VASP/Mena (EVMKO) KO (*32*) (P), EVM C5KO (Q to S), and EVM C5LKO (T to V) cells visualizing the actin cytoskeleton using fluorescent phalloidin. (**W**) Lamellipodium width of EVMKOs, EVM C5KOs, and EVM C5KOs. Kruskal-Wallis test combined with Dunn’s multiple comparison test, *n* = 50 cells for each experimental group, *P* values shown in the chart. Black lines indicate medians (0.8434, 0.8055, 0.7916, 0.7916, 0.7812, 0.878, and 0.8049 μm), and red lines indicate quartile ranges. Scale bars, 20 μm in standard panels and 2 μm in insets.

Instead, we asked whether ArpC5 and ArpC5L-containing lamellipodia might exhibit differences in actin filament elongation. We, therefore, hypothesized that ArpC5/5L isoform–specific Arp2/3 complexes might be able to regulate the localization and/or activity of actin filament elongating proteins. We thus tested for the presence of the Ena/VASP family proteins at the leading edge (*33*). Mena (Fig. 5, A to F) and VASP (Fig. 5, G to L) accumulated at the leading edge of B16-F16 WT (Fig. 5, A, B, G, and H) and C5LKO cells (Fig. 5, E, F, K, and L), but not in C5KO cells (Fig. 5, C, D, I, and J). Mena and VASP were still commonly found at the tips of finger-like protrusions in C5KO cells (Fig. 5D, inset). We corroborated these results via quantification (Fig. 5, M to O) and found the same pattern in Rat2 WT, C5KO, and C5LKO cells (fig. S15, A to N). Quantitative Western blotting further revealed that the decreased presence of Mena and VASP was not due to a reduction of their respective protein levels as they were largely comparable for WT, C5KO, and C5LKO of a given cell type (fig. S15O and table S1).

To further corroborate a connection between ArpC5 isoform specificity and changed accumulation of Ena/VASP family proteins at the leading edge, we generated genetic KOs of ArpC5 and ArpC5L in a recently published Ena/VASP/Mena triple-KO (EVMKO) B16-F1 line (*32*), resulting in EVM C5KO clones #7, #10, and #13, and EVM C5LKO clones #1, #2, and #5 (fig. S16). Visualization of the actin cytoskeleton by phalloidin staining revealed that lamellipodia widths of EVM C5KOs and EVM C5LKOs were indistinguishable from EVM-only KOs (Fig. 5, P to W). This clearly indicates that the lamellipodial phenotypes we observe upon removal of ArpC5 isoforms in WT cells are caused by the differential recruitment of Ena/VASP proteins to the leading edge, which, in turn, is dependent on the activity of individual ArpC5 isoforms.

Last, to verify that the reduced localization of Mena and VASP is a specific effect of incorporation of ArpC5 isoforms into branch junctions and not a mere consequence of the reduced lamellipodium phenotype itself, we tested for the localization of additional lamellipodial tip components determining protrusion and lamellipodia formation. We observed a very minor reduction of WAVE2 localization at the leading edges of both C5KO and C5LKO (fig. S17), in stark contrast to the almost complete reduction of Mena and VASP in C5KOs.

These results provide evidence that the reduced localization of Ena/VASP family members is not simply dependent on the extent of active lamellipodial protrusion. Instead, it suggests that isoform-specific Arp2/3 complex subpopulations are directly capable of affecting the distribution of specific downstream effectors to determine actin filament elongation.

## DISCUSSION

**Fig. 6.**
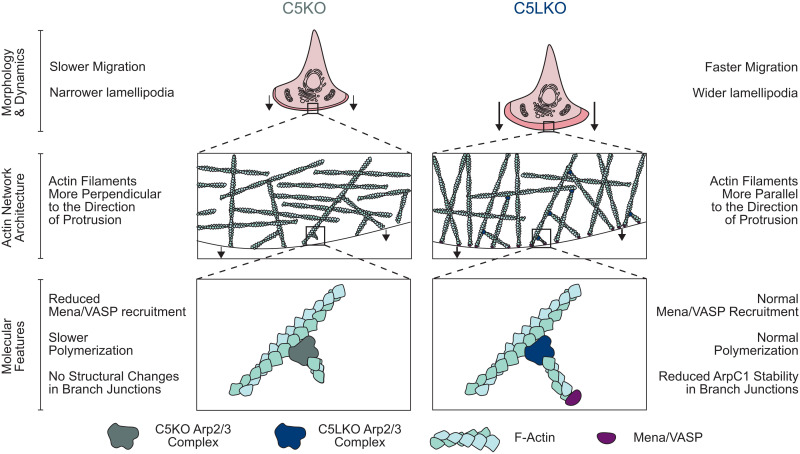
Schematic summary ArpC5 isoform–dependent regulation of lamellipodium characteristics and cell migration. Schematic representation of the phenotypes observed for C5KO and C5LKO cells and of how altered actin filament polymerization velocities caused by differential recruitment of Mena/VASP overcome the intrinsically different nucleation speeds of the associated Arp2/3 complexes.

We have used an integrated cell and structural biology approach to show on the cellular, ultrastructural, and molecular level that the two distinct ArpC5 isoforms affect cell migration differentially. We show that C5KO cells exhibit impaired motility and lamellipodia formation, strongly altered actin architecture, and reduced recruitment of actin filament elongators of the Ena/VASP family to the leading edge (Fig. 6). These results indicate that ArpC5 isoform composition can act as a regulator of actin polymerization efficiency in lamellipodia by altering the extent of Ena/VASP protein accumulation at the leading edge. Our findings further suggest that Ena/VASP-dependent elongation of ArpC5-containing Arp2/3 complex–nucleated filaments is able to overcome the reduced nucleation efficiency of ArpC5-containing Arp2/3 complexes, as originally reported by the Way laboratory (Fig. 6) (*17*, *19*). Note that ArpC5 expression and Ena/VASP expression are associated with increased metastasis and poorer overall survival in different cancers (*25*–*27*, *51*–*54*). To what extent this relates to the molecular findings in the context of ArpC5 isoform functions described here will have to be determined in future studies.

It is intriguing to speculate how an Arp2/3 complex of a given isoform composition is able to define the elongation properties of the filament it has nucleated. Does ArpC5 specifically support the delivery of Ena/VASP, or does ArpC5L prevent the association of these elongation factors with barbed actin filament ends? A previous in vitro study using tissue-derived Arp2/3 complex reported the importance of the source tissue of Arp2/3 complex, which can determine the effects of VASP on WAVE-dependent actin polymerization (*55*). In the presence of thymus-derived Arp2/3 complexes (high ArpC5/ArpC5L ratio), VASP boosted the otherwise low actin polymerization rate significantly via its capability to enhance filament elongation. In contrast, VASP lowered the polymerization of actin in the presence of brain-derived Arp2/3 complexes (low ArpC5/ArpC5L ratio), which appeared to have higher baseline nucleation activity. These findings suggest an antagonistic cross-regulation between ArpC5L and Ena/VASP proteins in WAVE-dependent actin networks, which is consistent with our observations. In addition, several studies also reported that the WRC directly interacts with Ena/VASP proteins (*55*–*57*). A similar interaction has not been described between the Arp2/3 complex and Ena/VASP family members. If WAVE complexes directly recruit filament elongators to nascent Arp2/3 complex–nucleated actin filaments, it is conceivable that the probability of Ena/VASP recruitment is increased with a longer residence time of the WRC at the Arp2/3 complex during activation and branch junction formation. The ArpC5-induced instability of ArpC1 (Fig. 3L), which harbors one part of the central high-affinity interaction site between NPF VCAs and the Arp2/3 complex (*58*), might cause less efficient Arp2/3-dependent nucleation and thus increased WRC residence times, which would, in turn, boost Ena/VASP recruitment. Such a mechanism could explain the observations made in our studies but remains speculative as further investigations are needed to fully unravel the cross-regulation between variable isoform-dependent Arp2/3 complex activities and the resulting downstream actin filament elongation of newly nucleated filaments.

Despite their isoform-specific differences, the shared functions of ArpC5 and ArpC5L are vital for Arp2/3 activity in cells, as we can show that lack of both isoforms results in complete loss of lamellipodia (fig. S3). In addition, earlier studies have shown the roles of ArpC5/5L in Arp2/3 complex assembly, stabilization, and nucleation activity (*17*, *19*, *43*). In vitro reconstitution experiments suggested that ArpC5/ArpC5L are involved in integrating ArpC1 into the complex (*43*). Our structures of isoform-specific branch junctions obtained from cells further emphasize such a functional connection between ArpC1 and ArpC5/ArpC5L, as we observe an increased flexibility of ArpC1 within ArpC5-containing Arp2/3 complex branch junctions. Nevertheless, we cannot comment on whether this affects either one or both of the ArpC1 isoforms present in the branch junction. Weakened interfaces between ArpC1 and ArpC5, caused by an ArpC5-specific linker sequence (fig. S13), could be causative for the observed increase in ArpC1 flexibility. However, the resolution of our structures is too low to unambiguously define the interactions that form the ArpC1/ArpC5 interface. A recent single-particle cryo-EM study solving structures of in vitro reconstituted, inactive Arp2/3 complexes at resolutions between 4 and 5 Å showed increased flexibility of ArpC5L compared to ArpC5. Nevertheless, this observation cannot fully explain altered ArpC1 stability because we observed stable ArpC1 in ArpC5L-containing branches. We also did not observe pronounced differences in the densities between ArpC5 and ArpC5L in our isoform-specific branch junction reconstructions from cells. This suggests that ArpC5 isoforms might be differentially stabilized in the inactive and active Arp2/3 complex and that the stabilization of ArpC1 might not be entirely dependent on ArpC5 isoforms. However, it remains to be clarified what exactly controls the stability of ArpC1 and how ArpC1 stability affects the branch junction as a whole. Future structural studies, either in vitro on reconstituted, active Arp2/3 complexes (*12*, *59*) or in situ, can provide further insights into how ArpC1 and ArpC5 isoform–specific branches are regulated by branch junction–stabilizing proteins, such as Cortactin, or branch junction–disassembling proteins, such as GMF (*60*) and Coronin 1b.

Our results show that the ArpC5 isoform is clearly associated with more prolific actin assemblies in lamellipodia of Rat2 fibroblasts and B16-F1 melanoma cells. These observations are in line with recently published work revealing that ArpC5 has the same effect in peripheral actin rings of CD4 T cells (*61*). This supports the notion that ArpC5/5L isoform–specific activities on cellular actin networks are generalizable over different cell types and species. This notwithstanding, ArpC5/5L isoforms showed cell type–specific differences in VACV actin tail formation in B16-F1 melanoma cells, Rat2 fibroblast, and HeLa cells. Specifically, in HeLa cells, the ArpC5/5L isoform–dependent actin tail length phenotype was inverted compared to B16-F1 and Rat 2 cells. We note that different cell lines can have different protein expression levels for the ArpC5 isoforms (figs. S2 and S6 and table S1). However, our observations that overexpression of either isoform is not able to rescue isoform-specific KO effects argue that the concentration of Arp2/3 complexes is not the main cause of the different VACV tail lengths in the cellular systems used. Further work is required to establish the molecular determinants that define VACV actin tail formation in different cell lines and species.

CRISPR-Cas9–mediated genome editing has revolutionized animal and plant cell biology and offers a substantial potential for cellular structural biology. In the present study, we were able to integrate structural and cell biology assays in one model system to obtain a better understanding of Arp2/3 complex regulation. Using cryo-ET and subtomogram averaging on genetically engineered cells, we determined structures of isoform-specific Arp2/3 complex branch junctions at ~8 Å resolution, revealing relevant differences. This study illustrates how cellular cryo-ET results can be complemented by a large number of experimental techniques applicable to cells. Furthermore, our work highlights the potential of cryo-ET to address challenging biological assemblies that are difficult to study with standard reductionist structural biology approaches (*62*).

## MATERIALS AND METHODS

### Antibodies and phalloidin

Primary antibodies were used in the following concentrations: Anti-ArpC5/C5L (Thermo Fisher Scientific no. PA5-30352) from rabbit, 1:5000 for Western blot; Anti-ArpC5 (Synaptic Systems no. 305 011) from mouse, 1:200 for immunostaining; Anti-ArpC5L (Abcam no. ab169763) from rabbit, 1:100 for immunostaining; Anti-ArpC1a (Abcam, no. ab133160) from goat, 1:2500 for Western blot; Anti-ArpC1b (Santa Cruz Biotechnology, no. sc-271342) from mouse, 1:250 for Western blot; Anti-Arp2 (Abcam, no. ab226476) from rabbit, 1:5000 for Western blot; Anti-Arp3 (Abcam, no. ab181164) from rabbit, 1:5000 for Western blot; Anti-Actin (Merck, no. A2066) from rabbit, 1:2500 for Western blot (fig. S7); Anti-Actin (Merck, no. A2228) from rabbit, 1:400 for immunostaining (fig. S8); Anti-FMNL2 (Abcam no. ab57963) from mouse, 1:1000 for Western blot and 1:50 for immunostaining (this antibody also interacts with FMNL3); Anti-Cortactin (Merck, no. 05-180) from mouse, 1:1000 for Western blot and 1:100 for immunostaining; Anti-B5 antibody [MAb VMC-20 (*63*, *64*), received from G. Cohen, University of Pennsylvania] from mouse, 1:100 for immunostaining; Anti-Mena (Merck, no. HPA028696) from rabbit, 1:2500 for Western blot and 1:200 for immunostaining; Anti-Cofilin (Santa Cruz Biotechnology, no. sc-376476) from mouse, 1:1000 for Western blot; Anti-p-Cofilin (Cell Signaling Technology, no. 3313) from rabbit, 1:1000 for Western blot; Anti-VASP (Merck, no. HPA005724) from rabbit, 1:2500 for Western blot and 1:200 for immunostaining; Anti-Actin (Merck, no. A1978) from mouse, 1:5000 for Western blot (fig. S10); Anti–glyceraldehyde-3-phosphate dehydrogenase (Abcam, no. ab125247) from mouse, 1:5000 for Western blot; Anti-Vinculin (Merck, no. V9131) from mouse, 1:5000 for Western blot; Peroxidase AffiniPure Goat Anti-Rabbit immunoglobulin G (IgG; Jackson Immuno Research no. 111-035-144), Peroxidase AffiniPure Goat Anti-Mouse IgG (Jackson Immuno Research no. 115-035-146), and Peroxidase AffiniPure Donkey Anti-Goat IgG (Jackson Immuno Research no. 705-035-003) were used in a dilution of 1:5000 as secondary antibodies for detection in Western blot applications. m-IgGk BP-CFL 488 (Santa Cruz Biotechnology, no. sc-516176) and Anti-Rabbit-IgG-Atto 594 (Merck, no. 77671-1ML-F) were used in 1:200 and 1:500 dilution, respectively, as secondary antibodies for immunostaining. Phalloidin-Atto488 was used complementary with Anti-Rabbit-IgG-Atto 594, and Phalloidin-Atto594 was used with m-IgGk BP-CFL 488 in 1:500 dilution to visualize actin filaments.

### Cloning dual expression plasmids

mRFP was amplified from aleu-mRFP (*65*) using GAGGACGTCGACATGGCCTCCTCCGAGGACGTCATCA/GTCCTCACTAGTTTATGCTCCAGTACTGTGGCGGCCC and inserted into the second multiple cloning site of PSF-CMV-CMV-SBFI-UB-PURO (Merck, no. OGS597-5UG) using Sal I/Spe I restriction digest and ligation. This yielded pCMV::empty_pCMV::RFP. ArpC5 and ArpC5L were amplified from a *Mus musculus* NIH-3T3 fibroblast (RRID:CVCL_0594) cDNA library using GAGAACACATGTCGAAGAACACGGTGTC/ATCGGGATCCCTACACGGTTTTCCTTGCAGT or GAGAACCCATGGCCCGGAACACACTGTC/GTCCTCGGATCCTTAAACAGTCTTTCTTGCTGTA, respectively. ArpC5 was digested using Pci I/Bam HI and inserted into the first multiple cloning site of Nco I/Bam HI–digested pCMV::empty_pCMV::RFP by ligation yielding pCMV::ArpC5_pCMV::RFP. ArpC5L was digested using Nco I/Bam HI and inserted into the first multiple cloning site of Nco I/Bam HI–digested pCMV::empty_pCMV::RFP by ligation yielding pCMV::ArpC5L_pCMV::RFP.

### Cell culture and KO line generation

WT *M. musculus* (Mm) B16-F1 (RRID: CVCL_0158) melanoma cells and *Rattus norvegicus* (Rn) Rat2 fibroblasts (RRID: CVCL_0513) were used in this study. B16-F1 WAVE1KO, WAVE2KO, WAVE1/2KO, and B16-F1 EVMKO cells were previously characterized (*32*, *34*). B16-F1 cells were cultured in Dulbecco’s modified Eagle medium GlutaMAX (Thermo Fisher Scientific, no. 31966047), supplemented with 10% (v/v) fetal bovine serum (Thermo Fisher Scientific, no. 10270106) and 1% (v/v) penicillin-streptomycin (Thermo Fisher Scientific, no. 15070063). Rat2 fibroblasts were cultured in the same medium with an added 1% (v/v) of 100× MEM Non-Essential Amino Acids Solution (Thermo Fisher Scientific, no. 11140068). Cells were incubated at 37°C and 5% CO_2_. Detachment of cells before seeding was performed with 0.05% (Thermo Fisher Scientific, no. 25300054) and 0.25% (Thermo Fisher Scientific, no. 25200056) trypsin for B16-F1 cells and Rat2 fibroblasts, respectively.

ArpC5 and ArpC5L genes were knocked out individually in B16-F1 (WT and EVMKO) and Rat2 cells by CRISPR-Cas9–mediated genome editing resulting in C5KO and C5LKO lines (*66*). The B16-F1 C5/C5L double KO lines were generated by disrupting ArpC5 in the B16-F1 C5LKO #16 background. The corresponding guide sequences GATATGACGAGAACAAGTTCG for MmArpC5, GATTCGTAGACGAGCACGAAG for MmArpC5L, GTCGTCGGCCCGCTTCCGGAAGG for RnArpC5, and GATCCACTCGGCGGAAGCGTGAGG for RnArpC5L were cloned into pSpCas9(BB)-2A-Puro (Addgene, ID 48139). Cells were transfected using Lipofectamine LTX Reagent with PLUS Reagent (Thermo Fisher Scientific, no. 15338100). Sixteen hours after transfection, cells were reseeded and enriched by selection in their respective media with an added 2.5 μg/ml of puromycin for B16-F1 and 3 μg/ml of puromycin (Merck no. p9620) for Rat2 cells, respectively, for 3 days. Surviving cells were diluted (~1:50) and incubated in nonselective media to allow for the formation of single cell–derived colonies. Approximately 7 days after seeding, individual colonies were selected and expanded for genotyping, first by Western blot, and then by genomic sequencing of the target loci (figs. S1, S2, and S16). For cell lines based on the EVMKO background, Tide-based sequence of polymerase chain reaction products was performed (*67*).

### Western blotting

Cyt-Ex buffer containing 22.5% (v/v) 4× Laemmli buffer (Bio-Rad, no. 1610747), 2.5% (v/v) 2-mercaptoethanol (Bio-Rad, no. 1610710), 75% (v/v) Cyt-Ex-Pre buffer [10 mM tris, 100 mM NaCl, 1 mM EDTA, 1 mM EGTA, 1% (v/v) Triton X-100, 0.1% (w/v) SDS, adjusted to pH 7.4], and cOmplete, EDTA-free protease inhibitor cocktail (Merck, no. 11873580001) was prepared shortly before sample extraction. Cells were washed with prechilled phosphate-buffered saline (PBS) and lysed with Cyt-Ex buffer on ice for 10 min before scraping and incubation at 95°C for 10 min. For estimating protein concentration, 10 μl of the sample was transferred into 100 μl of Amido-Black-Staining solution [0.25% (w/v) Amido black 10 B (Merck, no. 1011670025), 45% (v/v) MeOH, 45% (v/v) ddH_2_O, and 10% (v/v) glacial HOAc]. After sedimentation at 20,000*g* for 3 min, the supernatant was removed and replaced with 150 μl of Amido-Black-Destaining solution [45% (v/v) MeOH, 45% (v/v) ddH_2_O, and 10% (v/v) glacial HOAc]. After another round of sedimentation at 20,000*g* for 3 min, the supernatant was removed, and the sediment was dissolved in 200 μl of 0.2 M NaOH. One hundred microliters of the solution was then used for photometric concentration measurement at 600 nm of absorption using a TECAN Infinite F500 plate reader (Tecan). Sample volumes corresponding to ten micrograms of protein were run on SDS–polyacrylamide gel electrophoresis (SDS-PAGE) gels, transferred to polyvinylidene difluoride membranes (AL Labortechnik, 42514.01) via wet blotting, and blocked in blocking solution [4% (w/v) skim milk powder (Merck, no. 70166-500G) and 1% (w/v) bovine serum albumin (BSA; Merck, no. 10735078001)] in tris-based saline containing 0.05% Tween 20 (TBST) at room temperature (RT) for 1 hour. All subsequent washings were performed in TBST in three 5-min steps. Blots were washed and incubated in primary antibodies solved in Antibody-Dilution solution [2% (w/v) BSA and 0.02% (w/v) sodium azide in TBST] at 4°C overnight. Blots were washed again and incubated in secondary antibody diluted in blocking solution at RT for 45 min. Blots were washed one last time and then stored in TBS until detection. ECL1 solution {100 μl of 250 mM Luminol [Merck, no. A8511-5G, solved in dimethyl sulfoxide (DMSO)] and 44 μl of 90 mM p-coumaric acid (Merck, no. C9008-5G, solved in DMSO) dissolved in 1 ml 1 M tris (pH 8.5) and then brought to 10 ml with ddH_2_O] and ECL2 solution [6 μl 30% (v/v) H_2_O_2_ (Merck, #H1009) in 10 ml with ddH_2_O] were prepared directly before application. The chemiluminescence signals were developed in a 1:1 mixture of ECL1 and ECL2. Images were acquired using an Amersham Imager 600 (GE Healthcare). Uncropped versions of all Western blot results are represented in fig. S18. Quantification of Western blots was performed using Fiji v1.52p (*68*). Background intensity was subtracted, band intensities of proteins of interest were normalized, and relative protein levels in comparison to control (B16-F1 WT, Rat2 WT, or B16-F1 EVMKO, respectively) were calculated.

For the quantification of total actin content, the procedures stated above were altered to follow more the workflow described in (*69*): Cells were detached using 0.05% trypsin (Thermo Fisher Scientific, no. 25300054). One part of cell suspension was used for phalloidin staining as described in the “Immunofluorescence and phalloidin staining” section, while ~200,000 cells were sedimented and washed twice with prechilled PBS. The supernatant was aspirated as completely as possible, and cells were lysed in 100 μl of Cyt-Ex buffer on ice for 10 min before incubation at 95°C for 10 min. Ten microliters of the sample was run on SDS-PAGE. For quantification, background intensity was subtracted, and relative protein levels in comparison to B16-F1 WT were calculated.

### VACV infection assay

For VACV infection assays, we based our approach on a previously published protocol (*70*). Cells were infected with VACV (Western reserve strain, kindly provided by A. Bergthaler, Medical University of Vienna) for 1 hour, washed three times with PBS, and incubated in culture medium for another 7 hours. VACV-infected cells were fixed with prewarmed 4% paraformaldehyde (Merck, no. P6148) in PBS for 1 hour to ensure the inactivation of the virus. The cells were washed three times with PBS and subjected to staining with Phalloidin-Atto594 and a previously characterized monoclonal anti-B5 antibody (*63*, *64*) before embedding in ProLong Gold Antifade Mountant (Thermo Fisher Scientific, no. P36934).

### Immunofluorescence and phalloidin staining

For rescue experiments (Fig. 2), cells were transfected using 300 ng of dual expression plasmid and Lipofectamine LTX Reagent with PLUS Reagent (Thermo Fisher Scientific, no. 15338100) and incubated 16 hours before seeding. For immunofluorescence and phalloidin staining, cells were trypsinized and seeded onto coverslips that were coated with laminin (25 μg/ml; Merck, #11243217001) in laminin coating buffer (50 mM tris and 150 mM NaCl, pH 7.4) at RT for 1 hour. Cells were allowed to settle on coverslips for 16 hours at 37°C and 5% CO_2_ before they were either used for infection assays or directly fixed.

For fixation of non–VACV-infected samples, the medium was aspirated and immediately replaced by prewarmed 4% paraformaldehyde (Merck, no. P6148) in PBS. After 20 min of fixation, cells were washed twice with PBS before being permeabilized via incubation with 0.1% Triton X-100 in PBS for 1 min. Permeabilized cells were washed twice with PBS, subjected to the indicated staining, and embedded using ProLong Gold Antifade Mountant (Thermo Fisher Scientific, no. P36934).

Non–VACV-infected samples were imaged on a Zeiss Axio Imager (Zeiss) equipped with a CoolLED p300 SB light source (CoolLED) and an LCI Plan-Neofluar 63×/1.3 Imm Corr DIC M27 objective (Zeiss). VACV-infected samples were imaged on a Zeiss Axio Observer (Zeiss) equipped with 405-, 488-, 561-, and 640-nm laser lines and a Plan-APOCHROMAT 63×/1.4 Imm Corr DIC Oil M27 objective (Zeiss).

Conversion of image formats, generation of merges/overlays, and intensity and distance measurements were performed in Fiji v1.52p. For the quantification of total F-actin on a per-cell basis, cell circumferences were traced, and the integral fluorescence intensity within a cell was calculated (average intensity after subtracting background multiplied by area).

Specifically, for immunostainings of FMNL2 and FMNL3, cells were fixed in freshly prepared and prewarmed 2% paraformaldehyde and 0.3% picric acid (Applichem no. A2520) in PBS for 10 min. Fixatives were then exchanged with −20°C methanol, and cells were incubated for another 3 min before a stepwise rehydration using 100 mM glycine (Merck no. G8898) in PBS. FMNL2/3 immunostainings were imaged on Eclipse-Ti2 (Nikon) equipped with an Apo TIRF 100× Oil DIC N2 objective (Nikon) and operated with Nikon’s NIS-Elements AR imaging software.

### Cortactin knockdown

For the Cortactin knockdown experiments, cells were transfected in 96-well plate wells using either 25 nM siGENOME Non-Targeting Pool #2 (Horizon Discovery, no. D-001206-14-05) or 25 nM Cortactin small interfering RNA SMARTPool (Horizon Discovery, no. L-044721-00-0005) using 0.5 μl of DharmaFECT 1 Transfection Reagent in a total volume of 100 μl. A second round of transfection of the same cells was performed according to the same protocol after 24 hours. Cells were then incubated for 24 hours and subsequently seeded either for immunostaining onto coverslips that were coated with laminin (25 μg/ml) or for Western blot sample preparation into 12-well plate wells. Cells were allowed to settle for 24 hours before they were subjected to immunostaining or Western blot (for details, see above).

### Random migration assay

B16-F1 cells were seeded onto μ-slide eight-well glass-bottom microscopy chambers (Ibidi, no. 80807) coated with laminin (25 μg/ml). Live-cell time-lapse image series were acquired with an Eclipse-Ti2 (Nikon) equipped with a Plan Apo λ 20×/0.75 DIC air PFS objective (Nikon) and operated with Nikon’s NIS-Elements imaging software. A custom-made environment chamber was used to maintain constant conditions of 37°C and 5% CO_2_. DIC time-lapse images of randomly chosen cell-containing areas were acquired for 15 hours every 10 min. Trajectories of individual cells were tracked manually in Fiji v1.47v using the built-in Fiji Manual Tracking plugin. For deriving the average speed of individual cells, trajectories were analyzed using the Chemotaxis and Migration Tool (Ibidi, https://ibidi.com/chemotaxis-analysis/171-chemotaxis-and-migration-tool.html).

### Fluorescence recovery after photobleaching

B16-F1 cells were transfected with 0.5 μg of enhanced green fluorescent protein (EGFP)–β-actin and 1 μl of JetPrime transfection reagent (VWR, no. 114-07) per well in six-well plates according to the manufacturer’s instructions. Sixteen hours after transfection, cells were seeded onto μ-slide eight-well glass-bottom microscopy chambers (Ibidi, no. 80427) coated with laminin (25 μg/ml) as described in the “Immunofluorescence and phalloidin staining” section. Live-cell time-lapse image series were acquired with a 100×/1.4 apochromatic objective on an inverted Axio Observer (Zeiss) featuring an incubation chamber heated to 37°C (Warner instruments), a DG4 light source (Sutter instruments), and a CoolSnap HQ2 camera (Photometrics). Randomly chosen EGFP-actin–expressing cells with steadily protruding lamellipodia were imaged at 3 s per frame with 500-ms exposure time. After four to five frames, a rectangular region containing a subsection of the lamellipodium was bleached (see regions of interest in Fig. 4) using a 405-nm diode laser at 80-mW output power, controlled by a 2D-VisiFRAP Realtime scanner and driven by VisiView software (Visitron Systems). Individual recordings were analyzed using Fiji v2.1.0. Image stacks were resliced along a line in the bleached region perpendicular to the cell leading edge, creating a kymograph. Retrograde flow and protrusion rate values were obtained by calculating the speed of the recovering actin network moving back or forth, respectively. The total actin polymerization rate was determined by adding the absolute values of retrograde flow and protrusion rate. The ratio of protrusion to total actin polymerization is expressed as protrusion efficiency.

### Cryo–electron tomography

B16-F1 cells were trypsinized and seeded onto 200-mesh gold holey carbon grids (R2/2-2 C, Quantifoil Micro Tools) placed in three-dimensional (3D)–printed grid holders (*49*) for cryo-ET specimen preparation. Before seeding, grids were glow-discharged using an ELMO glow discharge unit (Cordouan Technologies) for 2 min and then coated with laminin (25 μg/ml) for 1 hour at RT. Cells were allowed to adhere to the grids at 37°C and 5% CO_2_ for 4 hours before they were extracted and fixed according to published protocols (*13*, *15*, *49*). In detail, grids were removed from grid holders and placed in 50 μl of drops of cytoskeleton buffer (10 mM MES, 150 mM NaCl, 5 mM EGTA, 5 mM glucose, and 5 mM MgCl_2_, pH 6.2) supplemented with 0.75% Triton X-100, 0.25% glutaraldehyde (Electron Microscopy Services, no. E16220), and phalloidin (0.1 μg/ml; Merck, no. P2141) for 1 min at RT. Grids were then transferred to 50-μl drops of cytoskeleton buffer containing 2% (w/v) glutaraldehyde and phalloidin (1 μg/ml) at RT for 15 min.

Subsequently, grids were vitrified using a Leica GP2 plunger (Leica Microsystems) with blotting chamber conditions of 80% humidity and 4°C. In the blotting chamber, the fixation solution was manually blotted and replaced with 3 μl of 10-nm colloidal gold coated with BSA suspended in PBS for WT or 3 μl of PBS for C5KO and C5LKO, respectively. After backside blotting for 3 s using the internal blotting sensor of the Leica GP2, the grids were vitrified in liquid ethane (−185°C) and then stored under liquid nitrogen conditions.

Tilt series were acquired under cryogenic conditions on a Thermo Fisher Scientific Krios G3i TEM equipped with a BioQuantum postcolumn energy filter and a K3 camera (Gatan), using SerialEM v3.8 (*71*). Acquisition of low- and medium-magnification maps allowed for defining areas of interest for subsequent high-resolution tomography data acquisition. Gain reference images were collected before data acquisition. DigitalMicrograph as integrated into the Gatan Microscopy Suite v3.3 (Gatan) and SerialEM were used for filter and microscope tuning. Tilt series were acquired with a filter slit width of 20 eV, and using a dose-symmetric tilt scheme (*72*) ranging from −66° to 66° with a 2° increment. The nominal defocus range was set to −1.5 to −4.5 μm and the nominal magnification was set to 53,000×, resulting in a pixel size of 1.693 Å. Tilt images were acquired as 5760 × 4092 pixel movies of eight frames. The dose per tilt was set to be 2.79 e/Å^2^, resulting in a calculated cumulative dose of 170 e/Å^2^ for the first 61 tilts per series. Datasets were acquired in separate acquisition sessions contributing 61 (WT), 97 (C5KO), and 61 (C5LKO) tilt series, respectively. For data acquisition settings, see fig. S12B.

### Cryo-ET data processing

Gain correction, frame alignment, and defocus estimation of individual tilts were performed in Warp v1.0.9 (*73*). Before exporting tilt series as .mrc stacks, poor-quality tilt-images caused, for example, by grid bars blocking the beam at high tilt angles were removed. Tilt-series alignment was done using patch tracking within the IMOD software package v4.11 (*74*).

Alignment parameters as determined in IMOD were imported into Warp. The metrics necessary for dose-dependent low-pass filtering were calculated during this step. Defocus parameters were then again determined for the whole tilt series. Eight times−binned (13.544 Å/px) tomograms were exported for template matching in the Dynamo package v1.1.333 (*75*). For template matching of branch junctions, a previously used template (*13*) was resampled to a pixel size of 13.544 Å/px using RELION v3.0.8 (*76*, *77*). For actin filaments, a template was generated from a model of aged, nucleotide-bound, and phalloidin-stabilized F-actin [PDB 6T20 from (*78*)] using the molmap function in chimera. The box of the resulting map was extended using RELION to be cube-shaped.

For cross-correlation calculation during template matching, masks consisting of two cylinders (140 Å radius) covering the branch or of one cylinder surrounding the single actin filament, respectively, were applied. Angular scanning around all three Euler angles was performed for a full 360° with a sampling step of 10°. False-positive cross-correlation peaks from predetermined areas containing gray value outliers were automatically removed. Subsequently, 400 positions from the branch junction search and up to 1000 positions from the actin filament search with the highest cross-correlation values per tomogram were selected for further processing.

Particles from different datasets were processed independently. Dynamo2m scripts v0.2.2 (*79*) were used to transform particle coordinates from Dynamo to RELION format. A total of 24,400 WT/38,800 C5KO/24,400 C5LKO branch junction–containing particles (30 px side length, 13.544 Å/px) and 61,061 WT/87,300 C5KO/54,900 C5LKO actin filament–containing particles (60 px side length, 6.722 Å/px) were exported using Warp. 3D classification in RELION was used to remove junk particles reducing the number of branch junction–containing particles to 9084 WT/15,339 C5KO/9831 C5LKO and actin filament–containing particles to 59,472 WT/85,966 C5KO/54,332 C5LKO.

Using Warp, all particles were then extracted into particles of 120 px side length with a pixel spacing of 3.386 Å. These datasets were then subjected to RELION 3D auto-refine using averages determined from the current particle orientations (low-pass filtered to 40 Å) as reference. Particles were automatically distributed into even and odd subsets during the RELION workflow. Subsequently, multiparticle refinement was performed in M v1.0.9 (*80*), simultaneously considering F-actin and branch junction particles. Tilt series were refined using image warp with a 9 × 6 grid and volume warp with a 4 × 6 × 2 × 10 grid, as well as tilt-angle optimization. Particle poses were refined for one temporal sampling point. Particles were reextracted from the refined tilt series at a pixel spacing of 1.693 Å and a side length of 200 px and were again aligned with RELION 3D auto-refine, using the result of the previous iteration filtered to 40 Å as reference. Multiparticle refinement was then again performed in M, simultaneously considering F-actin and branch junction particles. Tilt series were refined using image warp with a 9 × 6 grid and volume warp with a 4 × 6 × 2 × 10 grid, as well as tilt-angle optimization and frame alignment. Particle poses were refined for one temporal sampling point. The filtered and sharpened maps of the branch junctions were considered the final structures for their respective datasets. Resolution at the 0.143 FSC criterion was calculated with RELION postprocess using the half-maps from M as input. The masks used for FSC measurements are shown in fig. S12. The difference map was calculated using TEMPy: DiffMap (*81*) as implemented in the CCP-EM software suite v1.5 (*82*, *83*), using low-pass filtering of both maps to 8.1 Å, a fractional difference cutoff of 0.4, and dust filtering.

Molecular models and cryo-ET density maps were visualized in ChimeraX v1.1–1.4 (*84*). Alignment of ArpC5 and ArpC5L for mapping residue differences on PDB 7TPT (*12*) was performed using the Clustal Omega web tool (*85*).

### Statistical analysis

Statistical analyses were performed with Prism v9.0.2 (GraphPad Software). Differences were assessed by a nonparametric two-tailed Student’s *t* test (two groups) or a Kruskal-Wallis test followed by a Dunn’s multiple comparison test with the respective WT as reference (more than two groups). We considered *P* values smaller than 0.05 as significant. All cell biological experiments were performed in three biological replicates. Quantifications from ultrastructural analysis stem from a single data acquisition per genotype. Sample numbers, medians, and *P* values can be found in the figures and figure legends. Table S2 contains descriptive statistics, normality tests, and test statistics for all quantifications of cellular phenotypes.

## Notes

View/request a protocol for this paper from *Bio-protocol*.
