# Supplementary material for: ArpC5 isoforms regulate Arp2/3 complex–dependent protrusion through differential Ena/VASP positioning
Source: Sci Adv. 2023 Jan 20;9(3):eadd6495. doi: 10.1126/sciadv.add6495 (PMC9858492; doi:10.1126/sciadv.add6495)
Supplement: Supplementary file 1 — Figs. S1 to S18 Table S1 [file sciadv.add6495_sm.pdf]

Science Advances
AAAS

Supplementary Materials for

ArpC5 isoforms regulate Arp2/3 complex–dependent protrusion through differential Ena/VASP positioning

Florian Fäßler *et al.*

Corresponding author: Florian K.M. Schur, florian.schur@ist.ac.at

Sci. Adv. **9**, eadd6495 (2023)
DOI: 10.1126/sciadv.add6495

The PDF file includes:

Figs. S1 to S18
Table S1
Legend for table S2
Legends for movies S1 to S4

Other Supplementary Material for this manuscript includes the following:

Table S2
Movies S1 to S4

B16-F1 ArpC5 knockout lines

Reference: MmC5 WT
ATGTCGAAGAACACGGTGTCGTCGGCCCGCTTCCGGAAGGTGGACGTGGACGAATATGACGAGAACAAGTTCGTGGACGAGGAGGACGGC…
M S K N T V S S A R F R K V D V D E Y D E N K F V D E E D G …

MmC5KO #16
Allele 1, T insertion, frame shift starting from residue 25, STOP after residue 64, 17 of 17 reads
ATGTCGAAGAACACGGTGTCGTCGGCCCGCTTCCGGAAGGTGGACGTGGACGAATATGACGAGAACAAGTTTCGTGGACGAGGAGGACGGC…
M S K N T V S S A R F R K V D V D E Y D E N K F R G R G G R …

MmC5KO #17
Allele 1, T insertion, frame shift starting from residue 25, STOP after residue 64, 13 of 20 reads
ATGTCGAAGAACACGGTGTCGTCGGCCCGCTTCCGGAAGGTGGACGTGGACGAATATGACGAGAACAAGTTTCGTGGACGAGGAGGACGGC…
M S K N T V S S A R F R K V D V D E Y D E N K F R G R G G R …
Allele 2, T deletion, frame shift starting from residue 24, STOP after residue 50, 7 of 20 reads
ATGTCGAAGAACACGGTGTCGTCGGCCCGCTTCCGGAAGGTGGACGTGGACGAATATGACGAGAACAAGTCGTGGACGAGGAGGACGGC…
M S K N T V S S A R F R K V D V D E Y D E N K S W T R R T A …

MmC5KO #19
Allele 1, T insertion, frame shift starting from residue 25, STOP after residue 64, 7 of 18 reads
ATGTCGAAGAACACGGTGTCGTCGGCCCGCTTCCGGAAGGTGGACGTGGACGAATATGACGAGAACAAGTTTCGTGGACGAGGAGGACGGC…
M S K N T V S S A R F R K V D V D E Y D E N K F R G R G G R …
Allele 2, T deletion, frame shift starting from residue 24, STOP after residue 50, 5 of 18 reads
ATGTCGAAGAACACGGTGTCGTCGGCCCGCTTCCGGAAGGTGGACGTGGACGAATATGACGAGAACAAGTCGTGGACGAGGAGGACGGC…
M S K N T V S S A R F R K V D V D E Y D E N K S W T R R T A …
Allele 3 TC deletion, frame shift starting from residue 24, STOP after residue 63, 6 of 18 reads
ATGTCGAAGAACACGGTGTCGTCGGCCCGCTTCCGGAAGGTGGACGTGGACGAATATGACGAGAACAAGTGTGGACGAGGAGGACGGC…
M S K N T V S S A R F R K V D V D E Y D E N K C G R G G R R …

B16-F1 ArpC5L knockout lines

Reference: MmC5L WT
ATGGCCCGGAACACACTGTCCTCACGCTTCCGCCGCGTGGATATCGACGAATTTGACGAGAACAAATTCGTAGACGAGCACGAAGAGGCAGCGGCG…
M A R N T L S S R F R R V D I D E F D E N K F V D E H E E A A A …

MmC5LKO #15
Allele 1, C deletion, frame shift starting from residue 27, STOP after residue 43, 7 of 18 reads
ATGGCCCGGAACACACTGTCCTCACGCTTCCGCCGCGTGGATATCGACGAATTTGACGAGAACAAATTCGTAGACGAGCAGAAGAGGCAGCGGCG…
M A R N T L S S R F R R V D I D E F D E N K F V D E Q K R Q R …
Allele 2, G insertion, frame shift starting from residue 28, STOP after residue 88, 11 of 18 reads
ATGGCCCGGAACACACTGTCCTCACGCTTCCGCCGCGTGGATATCGACGAATTTGACGAGAACAAATTCGTAGACGAGCACGGAAGAGGCAGCGGCG…
M A R N T L S S R F R R V D I D E F D E N K F V D E H G R G S G …

MmC5LKO #16
Allele 1, G insertion, frame shift starting from residue 28, STOP after residue 88, 20 of 20 reads
ATGGCCCGGAACACACTGTCCTCACGCTTCCGCCGCGTGGATATCGACGAATTTGACGAGAACAAATTCGTAGACGAGCACGGAAGAGGCAGCGGCG…
M A R N T L S S R F R R V D I D E F D E N K F V D E H G R G S G …

MmC5LKO #20
Allele 1, G insertion, frame shift starting from residue 28, STOP after residue 88, 10 of 18 reads
ATGGCCCGGAACACACTGTCCTCACGCTTCCGCCGCGTGGATATCGACGAATTTGACGAGAACAAATTCGTAGACGAGCACGGAAGAGGCAGCGGCG…
M A R N T L S S R F R R V D I D E F D E N K F V D E H G R G S G …
Allele 2, C deletion, frame shift starting from residue 28, STOP after residue 43, 7 of 18 reads
ATGGCCCGGAACACACTGTCCTCACGCTTCCGCCGCGTGGATATCGACGAATTTGACGAGAACAAATTCGTAGACGAGCACGAGAGGCAGCGGCG…
M A R N T L S S R F R R V D I D E F D E N K F V D E H E R Q R …

B16-F1 ArpC5/ArpC5L double knockout lines

MmC5/C5LKO #2 (C5KO in C5LKO #16)
Allele 1, A insertion, frame shift starting from residue 24, STOP after residue 64, 5 of 20 reads
ATGTCGAAGAACACGGTGTCGTCGGCCCGCTTCCGGAAGGTGGACGTGGACGAATATGACGAGAACAAGATTCGTGGACGAGGAGGACGGC…
M S K N T V S S A R F R K V D V D E Y D E N K I R G R G G R …
Allele 2, T insertion, frame shift starting from residue 25, STOP after residue 64, 15 of 20 reads
ATGTCGAAGAACACGGTGTCGTCGGCCCGCTTCCGGAAGGTGGACGTGGACGAATATGACGAGAACAAGTTTCGTGGACGAGGAGGACGGC…
M S K N T V S S A R F R K V D V D E Y D E N K F R G R G G R …

MmC5/C5LKO #16 (C5KO in C5LKO #16)
Allele 1, GT deletion, frame shift starting from residue 24, STOP after residue 63, 5 of 20 reads
ATGTCGAAGAACACGGTGTCGTCGGCCCGCTTCCGGAAGGTGGACGTGGACGAATATGACGAGAACAATCGTGGACGAGGAGGACGGC…
M S K N T V S S A R F R K V D V D E Y D E N N R G R G G R …
Allele 2, C insertion, frame shift starting from residue 24, STOP after residue 64, 15 of 20 reads
ATGTCGAAGAACACGGTGTCGTCGGCCCGCTTCCGGAAGGTGGACGTGGACGAATATGACGAGAACAAGTCTCGTGGACGAGGAGGACGGC…
M S K N T V S S A R F R K V D V D E Y D E N K S R G R G G R …

MmC5/C5LKO #20 (C5KO in C5LKO #16)
Allele 1, T insertion, frame shift starting from residue 25, STOP after residue 64, 15 of 20 reads
ATGTCGAAGAACACGGTGTCGTCGGCCCGCTTCCGGAAGGTGGACGTGGACGAATATGACGAGAACAAGTTTCGTGGACGAGGAGGACGGC…
M S K N T V S S A R F R K V D V D E Y D E N K F R G R G G R …
Allele 2, 11 base pair deletion, frame shift starting from residue 20, STOP after residue 64, 5 of 20 reads
ATGTCGAAGAACACGGTGTCGTCGGCCCGCTTCCGGAAGGTGGACGTGGACGAATATGACTCGTGGACGAGGAGGACGGC…
M S K N T V S S A R F R K V D V D E Y R G R G G R …

Rat2 ArpC5 knockout lines

Reference: RnC5 WT

ATGTCGAAGAACACGGTGTCGTCGGCCCGCTTCCGGAAGGTGGACGTGGACGAATATGATGAGAACAAGTTCGTGGACGAGGAGGACGGC…
M S K N T V S S A R F R K V D V D E Y D E N K F V D E E D G …

RnC5KO #2
Allele 1, C insertion, frame shift starting from residue 12, STOP after residue 19, 18 of 31 reads
ATGTCGAAGAACACGGTGTCGTCGGCCCGCTTCCCGGAAGGTGGACGTGGACGAATATGATGAGAACAAGTTCGTGGACGAGGAGGACGG…
M S K N T V S S A R F P E G G R G R I STOP
Allele 2, C deletion, frame shift starting from residue 12, STOP after residue 50, 13 of 31 reads
ATGTCGAAGAACACGGTGTCGTCGGCCCGCTTCGGAAGGTGGACGTGGACGAATATGATGAGAACAAGTTCGTGGACGAGGAGGACGGC…
M S K N T V S S A R F G R W T W T N M M R T S S W T R R T …

RnC5KO #4
Allele 1, C deletion, frame shift starting from residue 12, STOP after residue 50, 30 of 30 reads
ATGTCGAAGAACACGGTGTCGTCGGCCCGCTTCGGAAGGTGGACGTGGACGAATATGATGAGAACAAGTTCGTGGACGAGGAGGACGGC…
M S K N T V S S A R F G R W T W T N M M R T S S W T R R T …

RnC5KO #8
Allele 1, G deletion, frame shift starting from residue 12, STOP after residue 50, 14 of 29 reads
ATGTCGAAGAACACGGTGTCGTCGGCCCGCTTCCGAAGGTGGACGTGGACGAATATGATGAGAACAAGTTCGTGGACGAGGAGGACGGC…
M S K N T V S S A R F R R W T W T N M M R T S S W T R R T …
Allele 2, CT insertion, frame shift starting from residue 12, STOP after residue 50, 15 of 29 reads
ATGTCGAAGAACACGGTGTCGTCGGCCCGCTTCCTCGGAAGGTGGACGTGGACGAATATGATGAGAACAAGTTCGTGGACGAGGAGGACGGC…
M S K N T V S S A R F L G R W T W T N M M R T S S W T R R T …

Rat2 ArpC5L knockout lines

Reference: WT RnC5L
ATGGCCCGGAACACACTGTCCTCACGCTTCCGCCGAGTGGATATCGACGAATTTGACGAGAACAAATTCGTAGACGAGCACGAAGAGGCGGCGGCGGCG…
M A R N T L S S R F R R V D I D E F D E N K F V D E H E E A A A A …

RnC5KO #11
Allele 1, G insertion, frame shift starting from residue 10, STOP after residue 18, 22 of 22 reads
ATGGCCCGGAACACACTGTCCTCACGGCTTCCGCCGAGTGGATATCGACGAATTTGACGAGAACAAATTCGTAGACGAGCACGAAGAGGCGGCGGCGGCG…
M A R N T L S S R L P P S G Y R R I STOP

RnC5KO #14
Allele 1, G insertion, frame shift starting from residue 10, STOP after residue 18, 9 of 22 reads
ATGGCCCGGAACACACTGTCCTCACGGCTTCCGCCGAGTGGATATCGACGAATTTGACGAGAACAAATTCGTAGACGAGCACGAAGAGGCGGCGGCGGCG…
M A R N T L S S R L P P S G Y R R I STOP
Allele 2, 11 base pair deletion, frame shift starting from residue 6, STOP after residue 14, 5 of 22 reads
ATGGCCCGGAACACGCTTCCGCCGAGTGGATATCGACGAATTTGACGAGAACAAATTCGTAGACGAGCACGAAGAGGCGGCGGCGGCG…
M A R N T L P P S G Y R R I STOP
Allele 3, major deletion including first 76 base pairs, 7 of 22 reads
No protein product
Allele 4, major deletion including first 424 base pairs, 1 of 22 reads
No protein product

RnC5KO #15
Allele 1, 11 base pair deletion, frame shift starting from residue 6, STOP after residue 14, 2 of 39 reads
ATGGCCCGGAACACGCTTCCGCCGAGTGGATATCGACGAATTTGACGAGAACAAATTCGTAGACGAGCACGAAGAGGCGGCGGCGGCG…
M A R N T L P P S G Y R R I STOP
Allele 2, major deletion including first 76 base pairs, 14 of 39 reads
No protein product
Allele 3, major deletion including first 114 base pairs, 19 of 39 reads
No protein product
Allele 4, major deletion including first 424 base pairs, 4 of 39 reads
No protein product

Figure S1: Sequencing results used to assess the ArpC5 and ArpC5L genotypes of cell lines used in this study

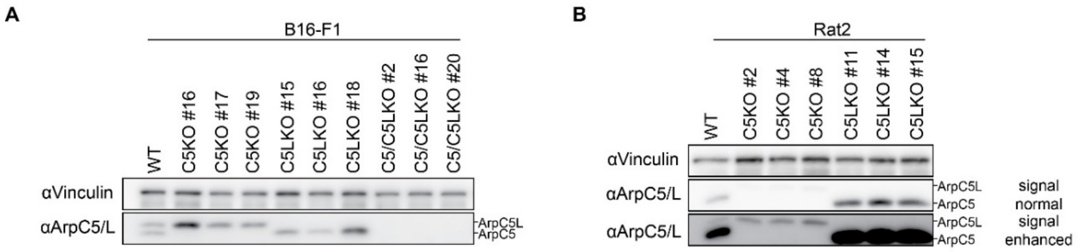

Figure S2: Western blot results used to assess the ArpC5 and ArpC5L genotypes of cell lines used in this study

(A, B) Representative Western blots showing levels of ArpC5 and ArpC5L proteins in three independent B16-F1 (A) and Rat2 (B) KO cell lines using a polyclonal antibody able to detect both isoforms. Double knockouts were only generated and analyzed in B16-F1 cells (A). Two visualizations of the same blot with different signal amplifications are shown for the Rat2 cells to allow visualization of both isoforms (B). Vinculin was used as loading control**.**

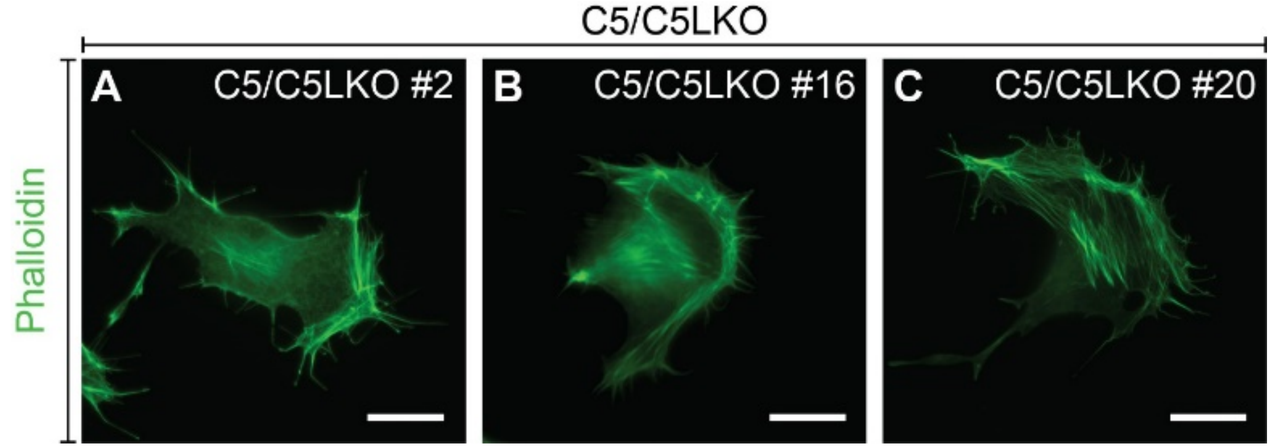

Figure S3: C5/C5LKO cells do not form any lamellipodia

(A-C) Representative epifluorescence micrographs of B16-F1 cells from three different C5/C5LKO lines visualizing the actin cytoskeleton using fluorescent phalloidin. All scale bars, 20µm.

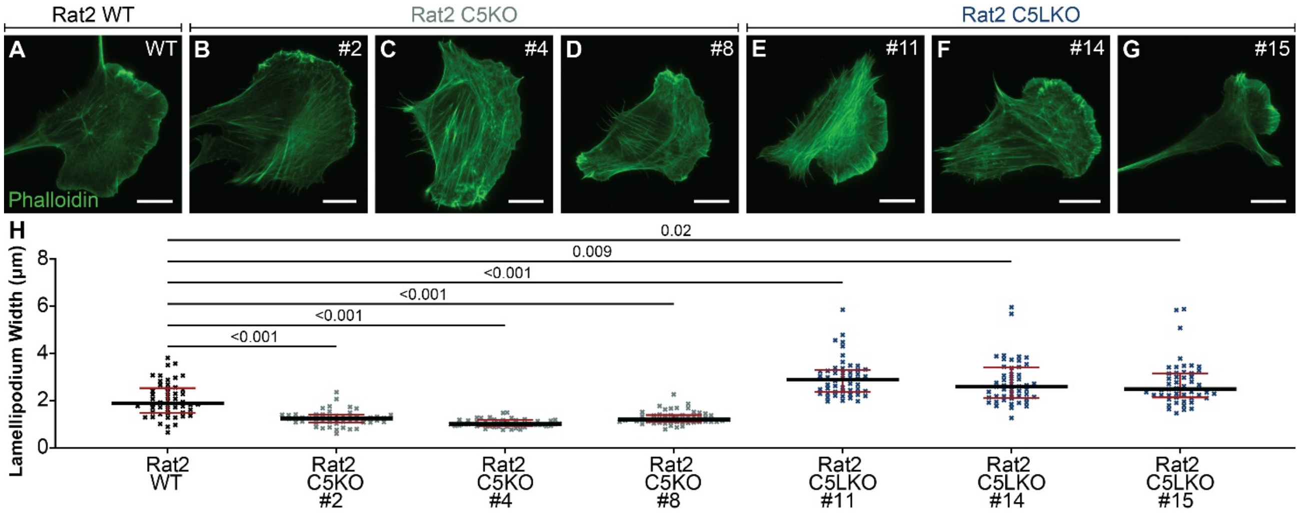

Figure S4: Rat2 C5KO and C5LKO cells recapitulate the lamellipodia phenotypes observed in the respective B16 knockout lines

(A-G) Representative epifluorescence micrographs of Rat2 wildtype (A), C5KO (B-D), and C5LKO (E-G) cells visualizing the actin cytoskeleton using fluorescent phalloidin. **(H)** Lamellipodium width of Rat2 C5KO and C5LKO lines and Rat2 wildtype cells. Kruskal-Wallis test combined with Dunn's multiple comparison test, n=50 cells for each experimental group, p values shown in the chart. Black lines indicate medians (1.899 µm, 1.253 µm, 1.016 µm, 1.211 µm, 2.894 µm, 2.607µm, 2.503µm), and red lines indicate quartile ranges. All scale bars, 20µm.

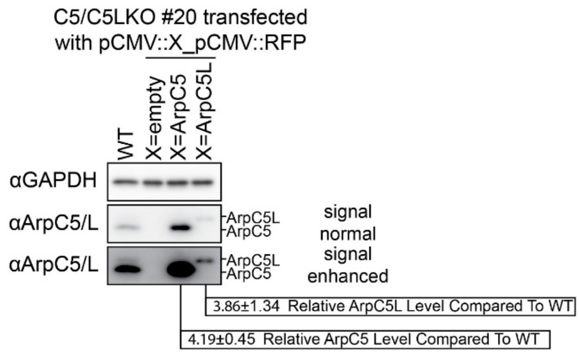

Figure S5: Expression levels of ArpC5 and ArpC5L in rescue experiments

Quantification of Western blots detecting ArpC5 and ArpC5L shows that the average abundance of these proteins per cell in the C5/C5LKO #20 background upon overexpression is ~4 times higher than in B16-F1 wildtype cells. GAPDH was used as loading control. Quantification of overexpression is related to data shown in Figure 2.

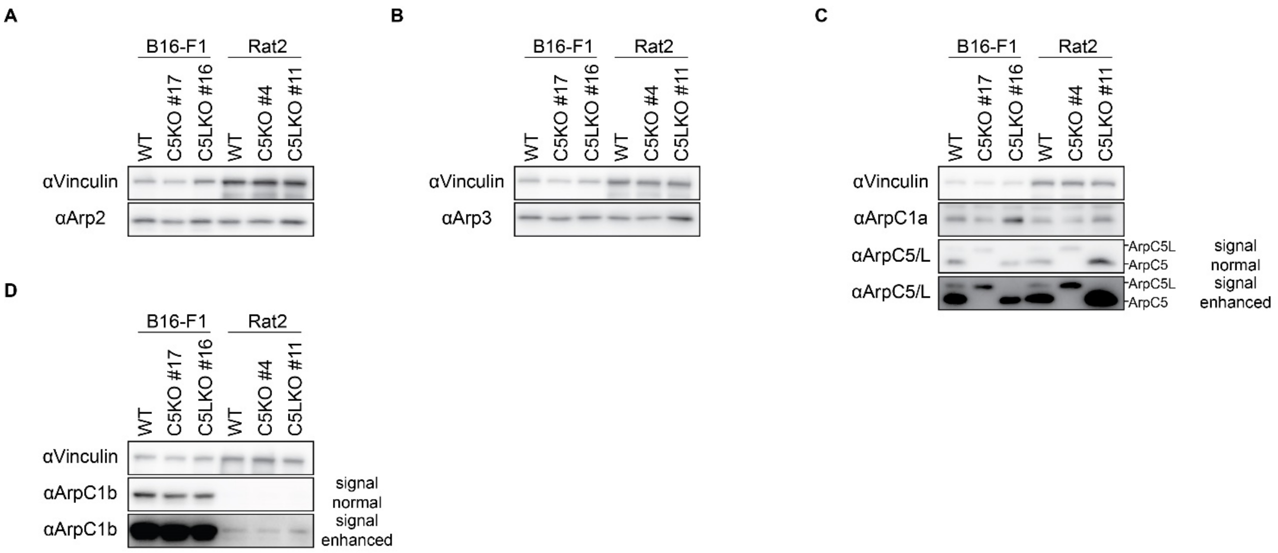

Figure S6: Expression levels of other Arp2/3 subunits

Western blot detecting **(A)** Arp2, **(B)** Arp3, **(C)** ArpC1a, and **(D)** ArpC1b confirms that abundance of these proteins is not severely reduced in B16-F1 and Rat2 C5KO and C5LKO cells compared to their respective wildtype counterparts. Two versions with different signal amplifications are shown for the ArpC1b and the polyclonal ArpC5/ArpC5L antibody to allow visualization in both cell types on the same membrane. Vinculin was used as loading control. Polyclonal ArpC5/ArpC5L antibody was employed to verify the sample genotypes. Quantification results are given in Table S1.

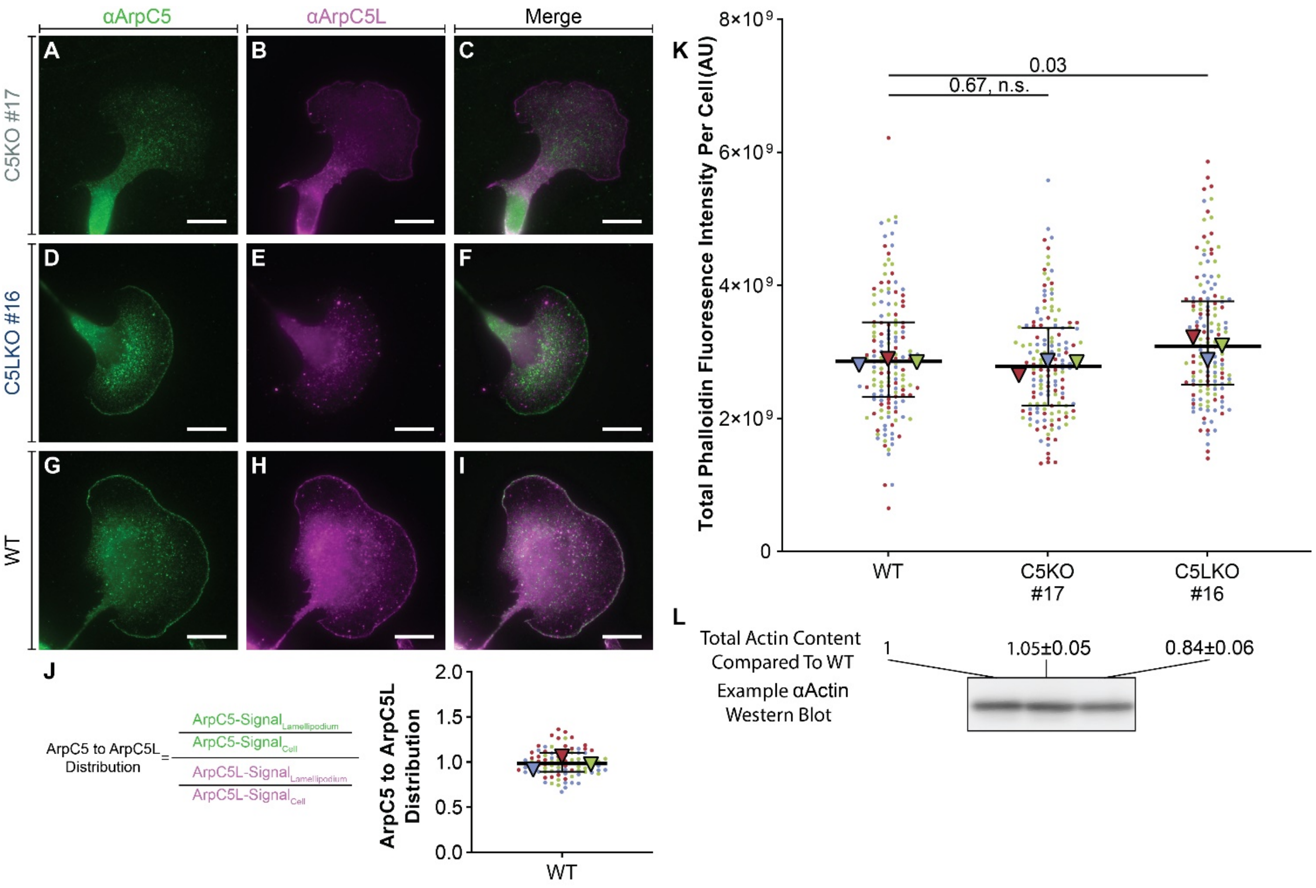

Figure S7: Immunofluorescence reveals similar ArpC5 and ArpC5L distribution in B16-F1 wildtype cells

(A-I) Representative epifluorescence micrographs of B16-F1 C5KO (A-C), C5LKO (D-F), and wildtype (G-I) cells visualizing the presence/absence and the localization of ArpC5 (A, D, G) and ArpC5L (B, E, I) via immunofluorescence, and showing the isoform specificity of the employed Arp5/ArpC5L antibodies. Merges are shown in (C, F, I). **(J)** ArpC5 to ArpC5L distribution was measured in B16-F1 wildtype cells using the displayed equation. If the ratio of ArpC5 intensity at the lamellipodium and within the cell body is exactly the same as the one of ArpC5L the expected result is 1. Pooled data from 3 independent experiments, n=150. Black lines indicate overall median (0.9851) and quartile ranges. **(K)** Total intensity of fluorescently labeled phalloidin was measured per cell as a proxy for the F-actin content in B16-F1 wildtype, C5KO, and C5LKO cells. Kruskal-Wallis test combined with Dunn's multiple comparison test on pooled data from 3 independent experiments, n=150, p values shown in the chart. Back lines indicate overall medians ($2.855x10^9$, $2.78x10^9$, $3.085x10^9$) and quartile ranges. AU, arbitrary units. **(L)** Quantitative analysis of actin Western blots was performed to estimate the ratio between the overall actin pool within B16-F1 wildtype, C5KO, and C5LKO cells. One representative blot is shown for illustration. Data points are color-coded according to the individual experiments. Triangles indicate the medians of the respective experiments. All scale bars, 20µm, n.s. = not significant.

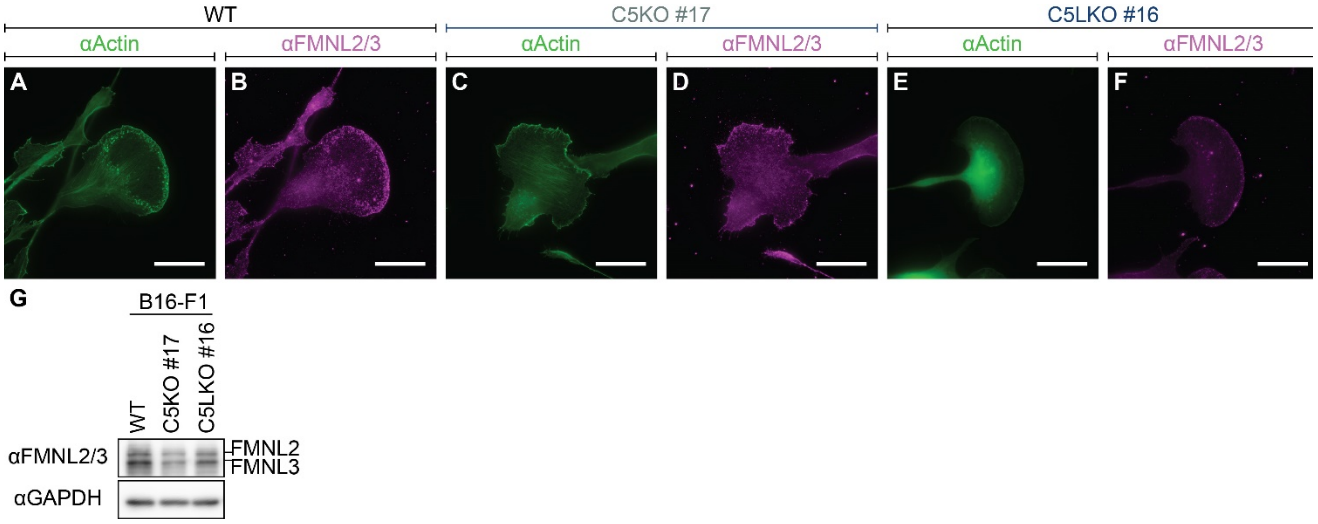

Figure S8: Expression of FMNL2 and -3 associated with the fingerlike protrusions found in C5KO is not upregulated in C5KO lines

(A-F) Representative epifluorescence micrographs of B16-F1 WT (A-C), C5KO (D-F), and C5LKO (G-I) cells visualizing the localization of actin (A, C, E) and FMNL2 and -3 (B, D, F) by immunofluorescence. **(G)** Western blot detecting FMNL2/3 confirms that protein levels are not upregulated in B16-F1 C5KO cells compared to the respective C5LKO and WT cells. GAPDH was used as loading control. Western blot quantification shown in Table S1. All scale bars, 20µm.

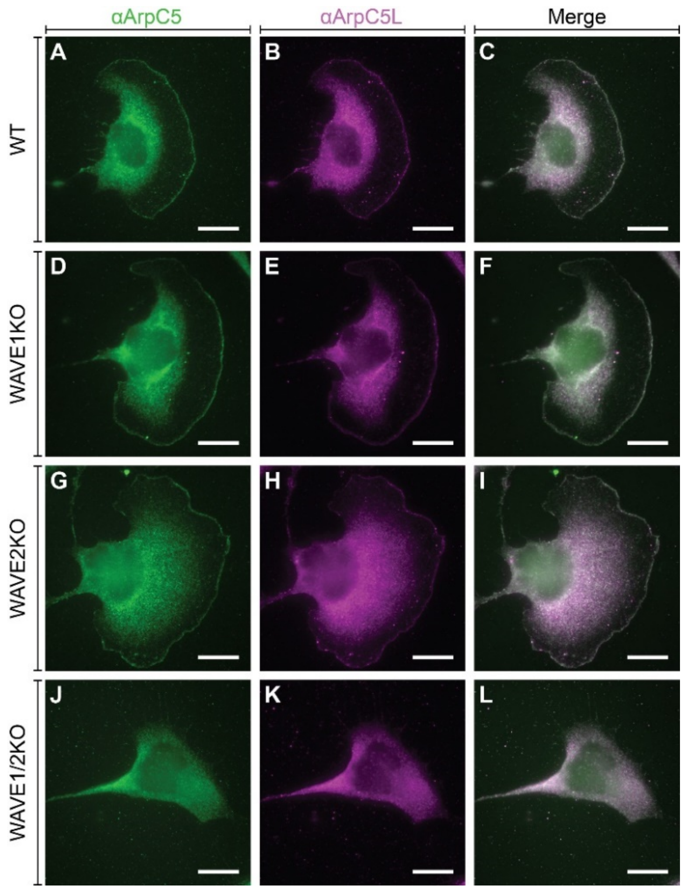

Figure S9: ArpC5 isoforms are not recruited by specific WAVE isoforms

(A-L) Representative epifluorescence micrographs of B16-F1 wildtype (A-C), WAVE1KO (D-F), WAVE2KO (G-I), and WAVE1/2KO (J-L) cells visualizing the presence/absence and the localization of ArpC5 (A, D, G, J), and ArpC5L (B, E, I, K) via immunofluorescence. Overlays of the signals are shown in (C, F, I, L). All scale bars, 20µm.

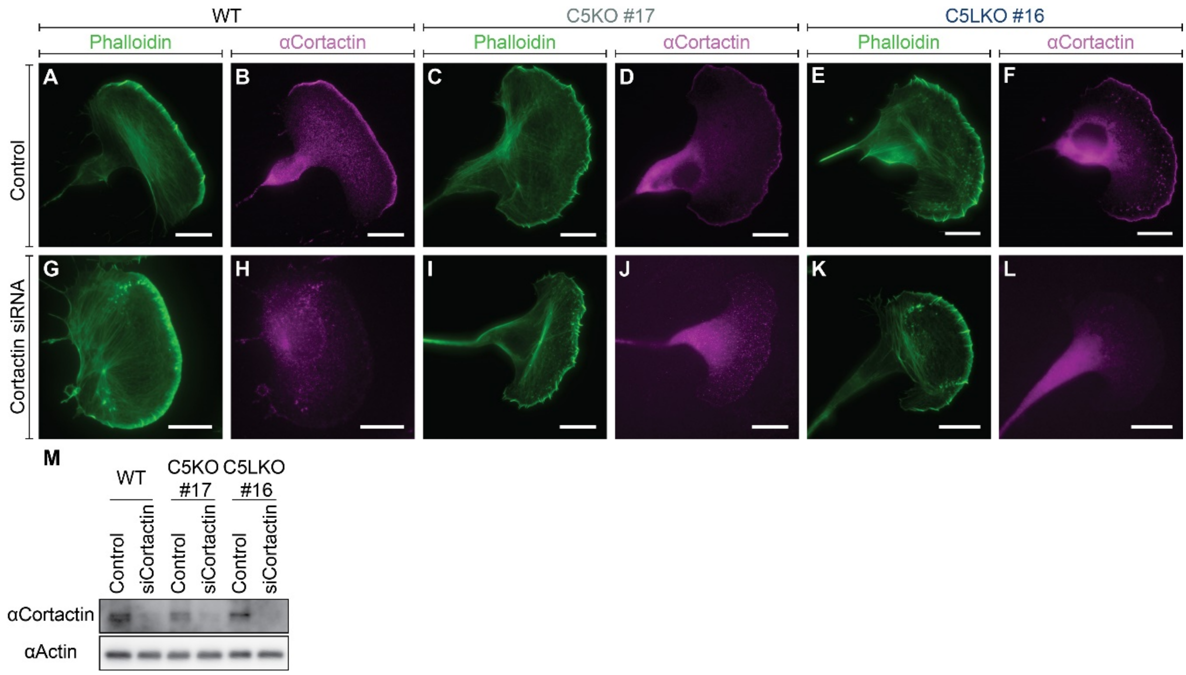

Figure S10: Cortactin knockdown does not interfere with the C5KO- and C5LKO-related lamellipodial phenotypes

(A-L) Representative epifluorescence micrographs of B16-F1 wildtype (A, B, G, H), C5KO (C, D, I, J), and C5LKO (E, F, K, L) cells after their transfection with control siRNA (A-F) or siRNA targeting Cortactin (G-L). The actin cytoskeleton is visualized by fluorescent phalloidin (A, C, E, G, I, K) and the presence/absence and the localization of Cortactin (B, D, F, H, J, L) via immunofluorescence. **(M)** Western blot analysis of a subpopulation of cells transfected together with the cells shown in (A-L) to verify Cortactin knockdown on a population scale. All scale bars, 20µm.

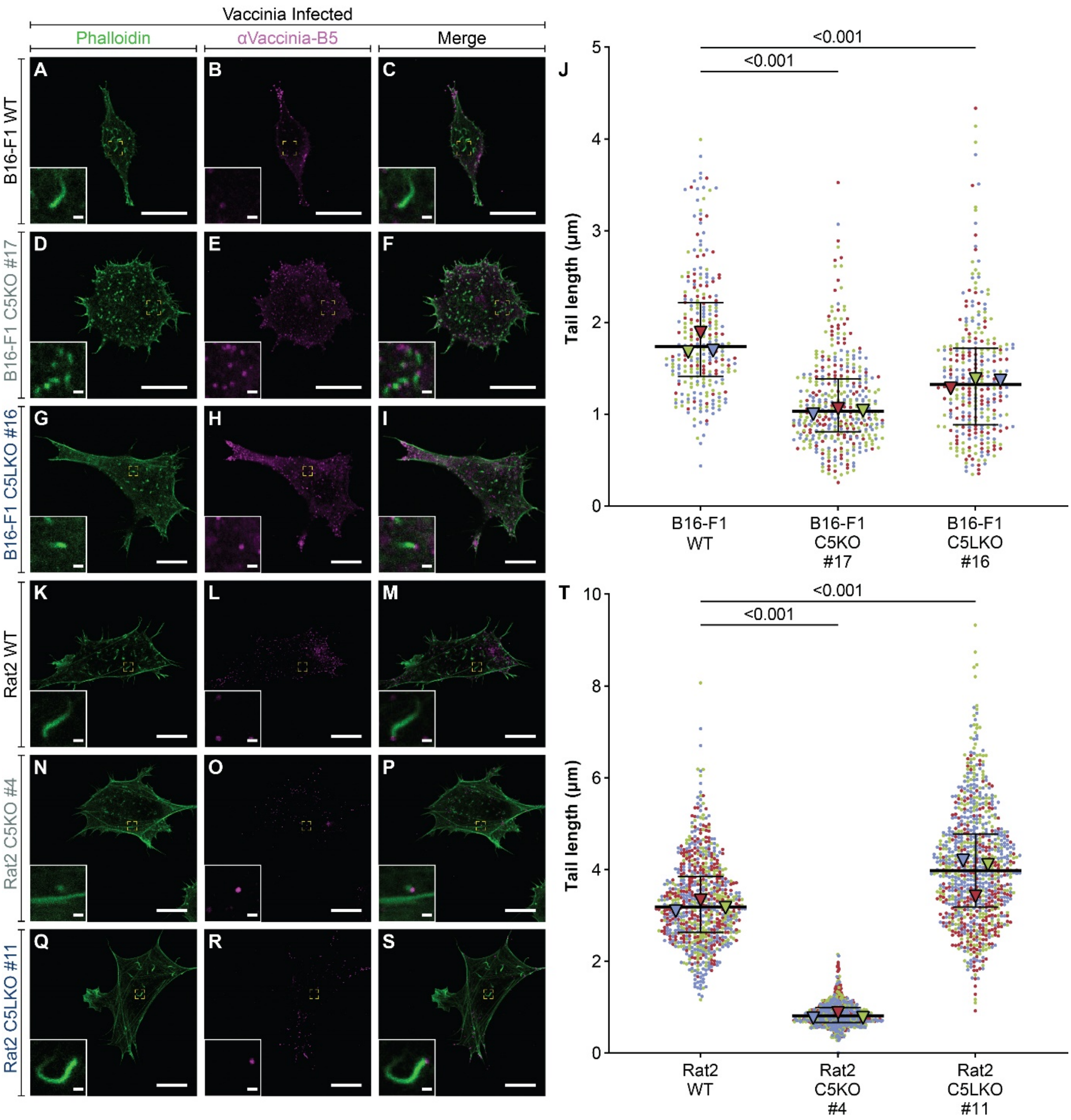

Figure S11: Effects on Vaccinia virus tails after loss of ArpC5 isoform

(A-I) Representative epifluorescence micrographs of Vaccinia virus-infected B16-F1 wildtype (A, B, C), C5KO (D, E, F), and C5LKO (G, H, I) cells visualizing the actin cytoskeleton and thus the Vaccinia tails using fluorescent phalloidin (A, D, G,) and the viruses using Vaccinia-B5 antibody (B, E, H). Overlays of the signals are shown in (C, F, I). Insets show individual viruses and their tails. Dashed yellow rectangles indicate their position in the micrographs. **(J)** Vaccinia tail lengths were measured in infected B16-F1 wildtype, C5KO, and C5LKO cells. Kruskal-Wallis test combined with Dunn's multiple comparison test on pooled data from 3 independent experiments, n=263, 391, 292, p values shown in the chart. Black lines indicate overall medians (1.739µm, 1.033µm, 1.,324µm) and quartile ranges. **(K-S)** Representative epifluorescence micrographs of Vaccinia virus-infected Rat2 wildtype (K, L, M), C5KO (N, O, P), and C5LKO (Q, R, S) cells visualizing the actin cytoskeleton and thus Vaccinia tails using the same staining as in (A-I). Insets show individual viruses and their tails. Their position in the micrographs is indicated by dashed yellow rectangles. **(T)** Vaccinia tail lengths were measured in infected Rat2 wildtype, C5KO, and C5LKO cells. Kruskal-Wallis test combined with Dunn's multiple comparison test on pooled data from 3 independent experiments, n=738, 728, 846, p values shown in the chart. Black lines indicate overall medians (3.183µm, 0.8095µm, 3.974µm) and quartile ranges. Scale bars, 20µm in standard panels and 1µm in enlarged insets. Data points are color-coded according to the individual experiments. Triangles indicate the medians of the respective experiments.

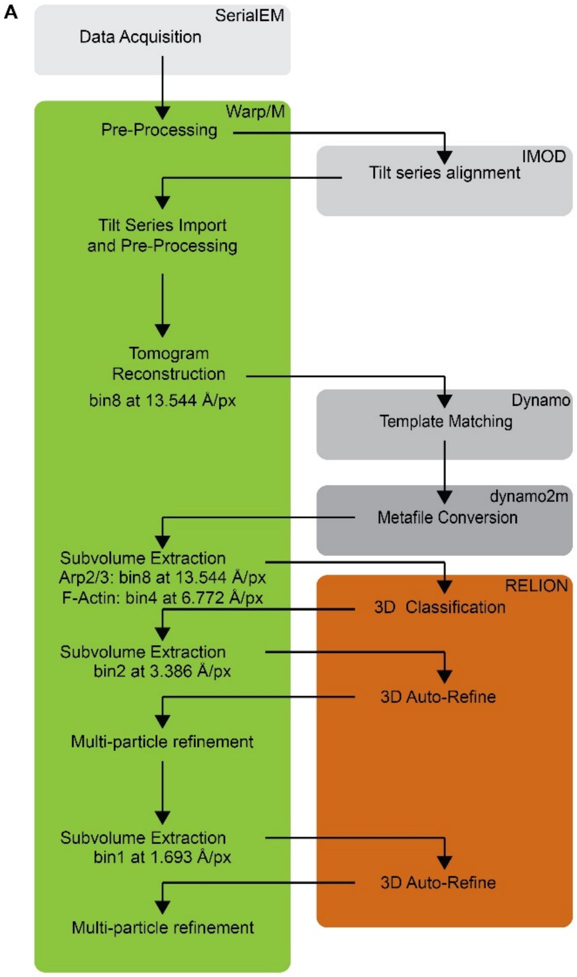

B

Genotype	WT	C5KO #17	C5LKO #16
Microscope	FEI Titan Krios 3Gi		
Voltage (keV)	300		
Detector	Gatan Bio Quantum K3		
Energy-Filter	Yes		
Slit Width (eV)	20		
Super-Resolution Mode	No		
Pixel size (Å/px)	1.693		
Defocus Range (µm)	-1.5 to -4.5	-1.5 to -4.0	
Defocus Step (µm)	0.25		
Acquisition Scheme	-66/66°, 2° dose-sym	-60/60°, 2° dose-sym	
Total Dose (e/Å²)	184	170	
Dose Rate (e/px/sec)	17.5	20.6	
Frames	8		
Tilt Series	61	97	61
Particles after Template Matching	24,400	38,800	24,400
Particles after Classification	9,084	15,339	9,831
Resolution (Å)	8.0	8.1	7.8

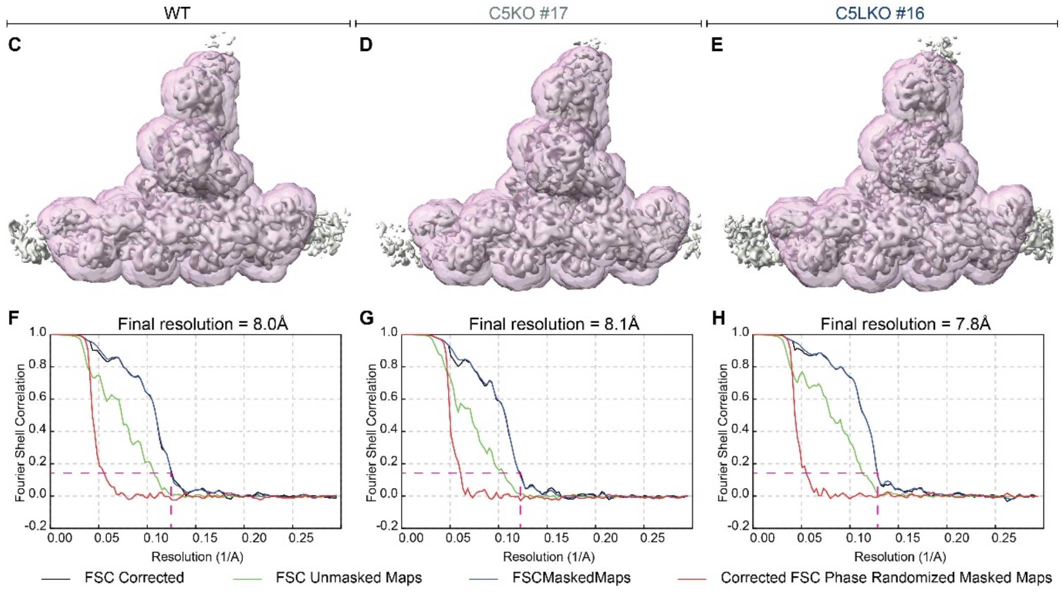

Figure S12: Workflow of image processing and resolution estimation for branch junction structures

(A) Flow chart indicating the data processing steps involved in generating the three structures of the active Arp2/3 complexes within respective branch junctions. Colored boxes indicate the use of specific software packages. **(B)** Data acquisition and image processing parameters. **(C-E)** Isosurface representation of the actin filament Arp2/3 complex branch junction in B16-F1 wildtype, C5KO, and C5LKO cells (shown in solid grey) filtered to their respective resolution determined by gold standard FSC calculation. Masks (transparent purple) used for FSC calculations are superimposed onto the respective structures. **(F-H)** FSC curves with indicated resolution cut-offs according to the 0.143 criterion.

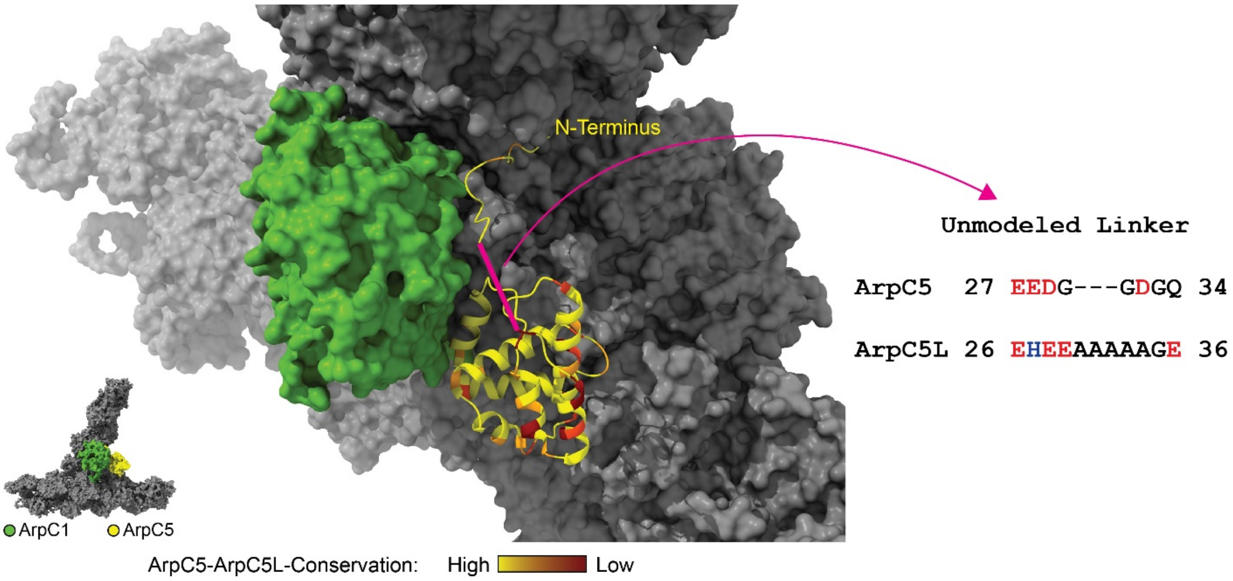

Figure S13: A non-modeled linker region proximal to ArpC1 harbors the largest differences between the primary structures of ArpC5 and ArpC5L
ArpC5 is color-coded according to the sequence homology between ArpC5 and ArpC5L. This reveals that most of the interaction surface with ArpC1 is highly conserved for those proteins. Sequence alignment of a non-modeled linker likely contacting ArpC1 is shown on the right. Residues with positively charged side chains are given in blue, and residues with negatively charged side chains are given in red. The molecular model of the branch junction used for representation is derived from PDB 7TPT (*12*).

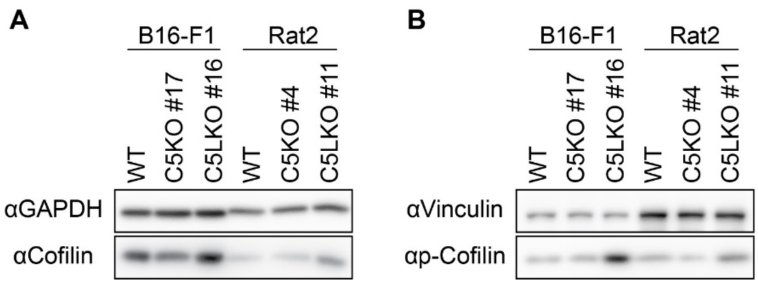

Figure S14: Cofilin and phospho-Cofilin are upregulated in C5LKO cells

(A-B) Western blots detecting Cofilin and p-Cofilin indicate altered levels of both in C5KO and C5LKO cells. GAPDH was used as loading control. Western blot quantification shown in Table S1.

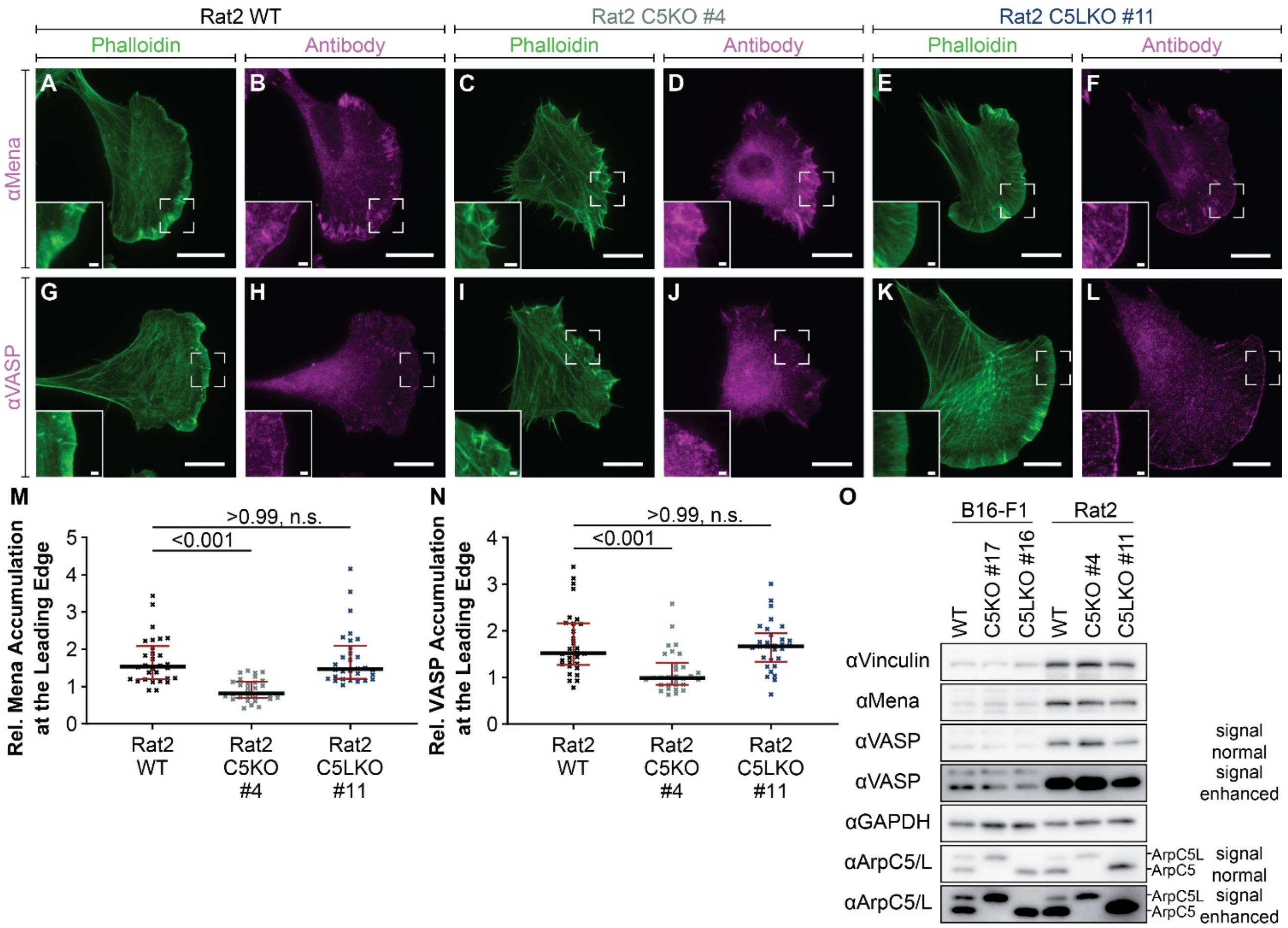

Figure S15: Rat2 C5KO and C5LKO cells recapitulate the Mena/VASP recruitment phenotypes observed in the respective B16 knockout lines

(A-L) Representative epifluorescence micrographs of Rat2 wildtype (A, B, G, H), C5KO (C, D, I, J), and C5LKO (E, F, K, L) cells visualizing the actin cytoskeleton using fluorescent phalloidin (A, C, E, G, I, K) and the localization of Mena (B, D, F) and VASP (H, J, L) via immunofluorescence. Insets show magnified areas indicated in the respective panels. **(M-N)** Quantitative analysis of the relative accumulation of Mena (M)/VASP (N) at the leading edges of Rat2 wildtype, C5KO, and C5LKO cells. Kruskal-Wallis test combined with Dunn's multiple comparison test, n=30 cells for each experimental condition, for both Mena and VASP, p values shown in the chart. Black lines indicate medians (Mena (panel M): 1.54, 0.8214, 1.47; VASP (panel N): - 1.521, 0.9927, 1.672) and red lines quartile ranges. **(O)** Western blot detecting Mena and VASP confirms that their absence at the leading edge in B16-F1 and Rat2 C5KO cells is due to reduced recruitment and not due to reductions at the protein level. Two versions with different signal amplifications are shown for the VASP and the polyclonal ArpC5/ArpC5L antibodies to allow visualization in both cell types on the same membrane. Vinculin and GAPDH were used as loading controls. Polyclonal ArpC5/ArpC5L antibody was employed to verify the sample genotypes. Scale bars, 20µm in standard panels and 2µm in enlarged insets, n.s. = not significant. Western blot quantification shown in Table S1

A

<u>B16-F1 EVM ArpC5 knockout lines</u>

```
Reference: MmEVMKO
ATGTCGAAGAACACGGTGTCGTCGGCCCGCTTCCGGAAGGTGGACGTGGACGAATATGACGAGAACAAGTTCGTGGACGAGGAGGACGGC…
 M  S  K  N  T  V  S  S  A  R  F  R  K  V  D  V  D  E  Y  D  E  N  K  F  V  D  E  E  D  G …

MmEVMC5KO #7
Allele 1, T insertion, frame shift starting from residue 25, STOP after residue 64, 98.4%
ATGTCGAAGAACACGGTGTCGTCGGCCCGCTTCCGGAAGGTGGACGTGGACGAATATGACGAGAACAAGTTTCGTGGACGAGGAGGACGGC…
 M  S  K  N  T  V  S  S  A  R  F  R  K  V  D  V  D  E  Y  D  E  N  K  F  R  G  R  G  G  R …

MmEVMC5KO #10
Allele 1, T insertion, frame shift starting from residue 25, STOP after residue 64, 45.6%
ATGTCGAAGAACACGGTGTCGTCGGCCCGCTTCCGGAAGGTGGACGTGGACGAATATGACGAGAACAAGTTTCGTGGACGAGGAGGACGGC…
 M  S  K  N  T  V  S  S  A  R  F  R  K  V  D  V  D  E  Y  D  E  N  K  F  R  G  R  G  G  R …
Allele 2 GT deletion, frame shift starting from residue 23, STOP after residue 63, 51.1%
ATGTCGAAGAACACGGTGTCGTCGGCCCGCTTCCGGAAGGTGGACGTGGACGAATATGACGAGAACAATCGTGGACGAGGAGGACGGC…
 M  S  K  N  T  V  S  S  A  R  F  R  K  V  D  V  D  E  Y  D  E  N  N  R  G  R  G  G  R …

MmEVMC5KO #13
Allele 1, T insertion, frame shift starting from residue 25, STOP after residue 64, 64%
ATGTCGAAGAACACGGTGTCGTCGGCCCGCTTCCGGAAGGTGGACGTGGACGAATATGACGAGAACAAGTTTCGTGGACGAGGAGGACGGC…
 M  S  K  N  T  V  S  S  A  R  F  R  K  V  D  V  D  E  Y  D  E  N  K  F  R  G  R  G  G  R …
Allele 2, T deletion, frame shift starting from residue 24, STOP after residue 50, 33.5%
ATGTCGAAGAACACGGTGTCGTCGGCCCGCTTCCGGAAGGTGGACGTGGACGAATATGACGAGAACAAGTCGTGGACGAGGAGGACGGC…
 M  S  K  N  T  V  S  S  A  R  F  R  K  V  D  V  D  E  Y  D  E  N  K  S  W  T  R  R  T  A …
```

<u>B16-F1 EVM ArpC5L knockout lines</u>

```
Reference: MmEVMKO
ATGGCCCGGAACACACTGTCCTCACGCTTCCGCCGCGTGGATATCGACGAATTTGACGAGAACAAATTCGTAGACGAGCACGAAGAGGCAGCGGCG…
 M  A  R  N  T  L  S  S  R  F  R  R  V  D  I  D  E  F  D  E  N  K  F  V  D  E  H  E  E  A  A  A …

MmEVMC5LKO #1
Allele 1, G insertion, frame shift starting from residue 28, STOP after residue 88, 98.2%
ATGGCCCGGAACACACTGTCCTCACGCTTCCGCCGCGTGGATATCGACGAATTTGACGAGAACAAATTCGTAGACGAGCACGGAAGAGGCAGCGGCG…
 M  A  R  N  T  L  S  S  R  F  R  R  V  D  I  D  E  F  D  E  N  K  F  V  D  E  H  G  R  G  S  G …

MmEVMC5LKO #2
Allele 1, G insertion, frame shift starting from residue 28, STOP after residue 88, 69.6%
ATGGCCCGGAACACACTGTCCTCACGCTTCCGCCGCGTGGATATCGACGAATTTGACGAGAACAAATTCGTAGACGAGCACGGAAGAGGCAGCGGCG…
 M  A  R  N  T  L  S  S  R  F  R  R  V  D  I  D  E  F  D  E  N  K  F  V  D  E  H  G  R  G  S  G …
Allele 2, AA deletion, frame shift starting from residue 28, STOP after residue 88, 29.2%
ATGGCCCGGAACACACTGTCCTCACGCTTCCGCCGCGTGGATATCGACGAATTTGACGAGAACAAATTCGTAGACGAGCACGGAGGCAGCGGCG…
 M  A  R  N  T  L  S  S  R  F  R  R  V  D  I  D  E  F  D  E  N  K  F  V  D  E  H  G  G  S  G …

MmEVMC5LKO #5
Allele 1, G insertion, frame shift starting from residue 28, STOP after residue 88, 75.1%
ATGGCCCGGAACACACTGTCCTCACGCTTCCGCCGCGTGGATATCGACGAATTTGACGAGAACAAATTCGTAGACGAGCACGGAAGAGGCAGCGGCG…
 M  A  R  N  T  L  S  S  R  F  R  R  V  D  I  D  E  F  D  E  N  K  F  V  D  E  H  G  R  G  S  G …
Allele 2, GG insertion, frame shift starting from residue 28, STOP after residue 44, 24%
ATGGCCCGGAACACACTGTCCTCACGCTTCCGCCGCGTGGATATCGACGAATTTGACGAGAACAAATTCGTAGACGAGCACGGGAAGAGGCAGCGGCG…
 M  A  R  N  T  L  S  S  R  F  R  R  V  D  I  D  E  F  D  E  N  K  F  V  D  E  H  G  K  R  Q  R …
```

B

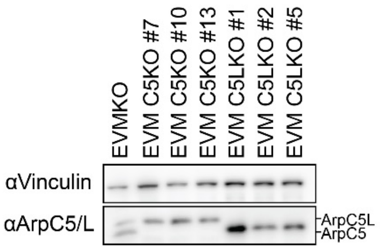

Figure S16: Assessment of the ArpC5 and ArpC5L genotypes of B16-F1 EVMKO cells by sequencing and Western blotting

(A) Sequencing results. **(B)** Representative Western blot showing levels of ArpC5 and ArpC5L proteins in three independent B16-F1 EVMKO-based cell lines per protein using a polyclonal antibody able to detect both isoforms, confirming successful KO.

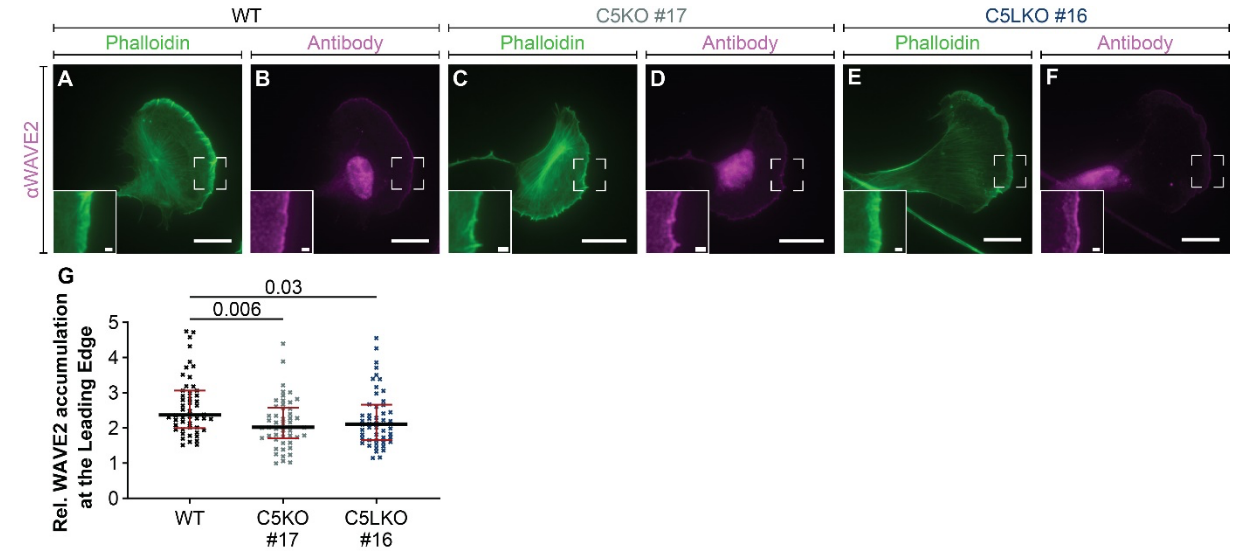

Figure S17: Loss of either ArpC5 isoform marginally reduces WAVE2 accumulation at the leading edge of protruding lamellipodia

(A-F) Representative epifluorescence micrographs of B16-F1 wildtype (A, B), C5KO (C, D), and C5LKO (E, F) cells visualizing the actin cytoskeleton using fluorescent phalloidin (A, C, E) and the localization of WAVE2 (B, D, F) by immunofluorescence. Insets show magnified areas indicated in the respective panels. **(G)** Quantitative analysis of the relative accumulation of WAVE2 at the leading edges of B16-F1 wildtype, C5KO, and C5LKO cells. Kruskal-Wallis test combined with Dunn's multiple comparison test, n=50 cells for each experimental condition, for both Mena and VASP, p values shown in the chart. Black lines indicate medians (2.367, 2.019, 2.110), and red lines quartile ranges. Scale bars, 20µm in standard panels and 1µm in insets.

Figure S2A

αVinculin

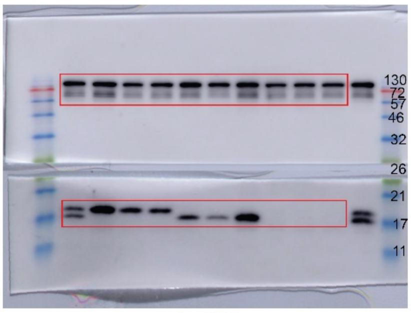

αArpC5/L

Figure S2B

αVinculin

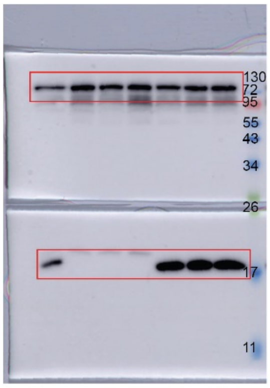

αArpC5/L

Figure S5

αGAPDH

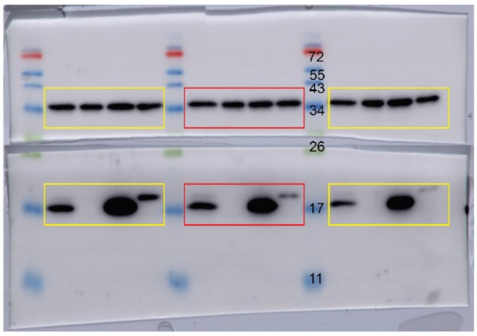

αArpC5/L

Figure S6A-B

αVinculin

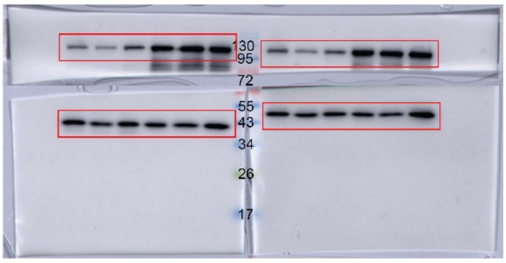

αArp2 αArp3

αVinculin

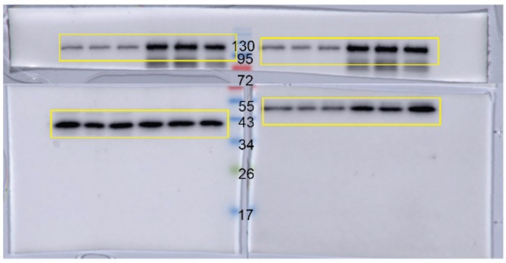

αArp2 αArp3

αVinculin

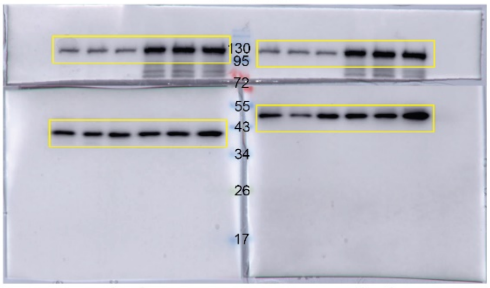

αArp2 αArp3

Figure S6C

αVinculin

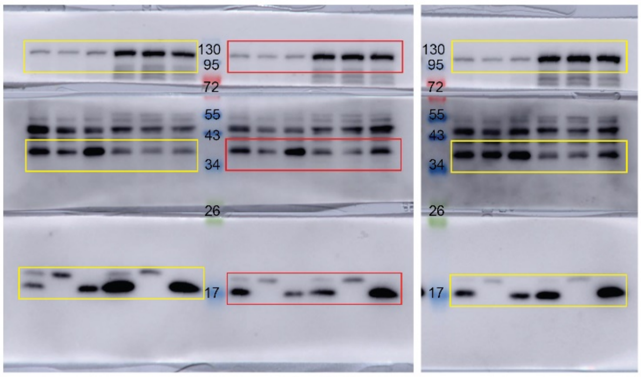

αArpC1a

αArpC5/L

Figure S6D, experiment was performed on the same blot as Figure S14B

αVinculin

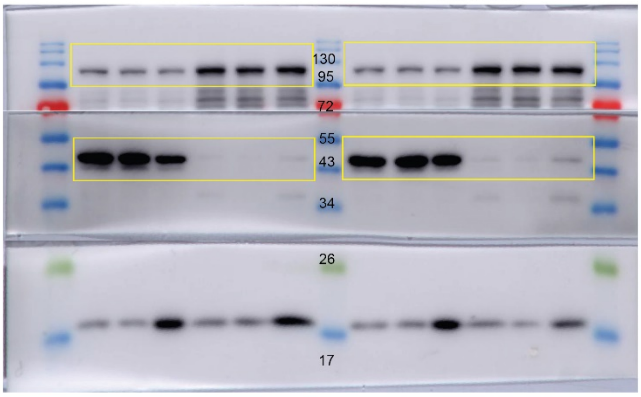

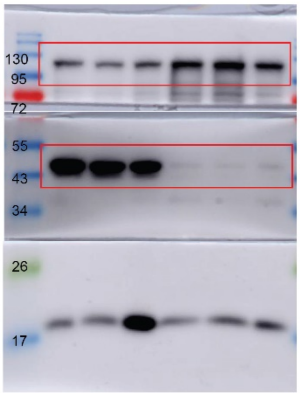

αArpC1b

αp-Cofilin (only used for Figure S14B)

Figure S7L

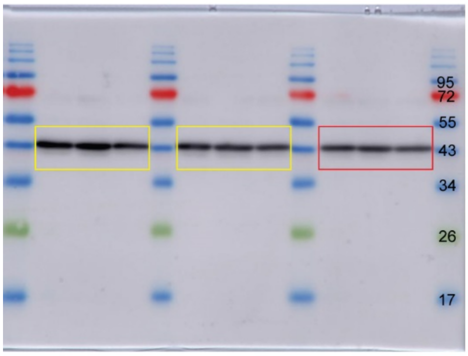

αActin

Figure S 10M

αCortactin

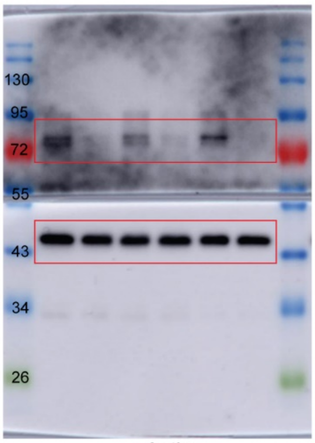

αActin

Figure S 8G, experiment was performed on the same blot as Figure S14A

αFMNL2/3

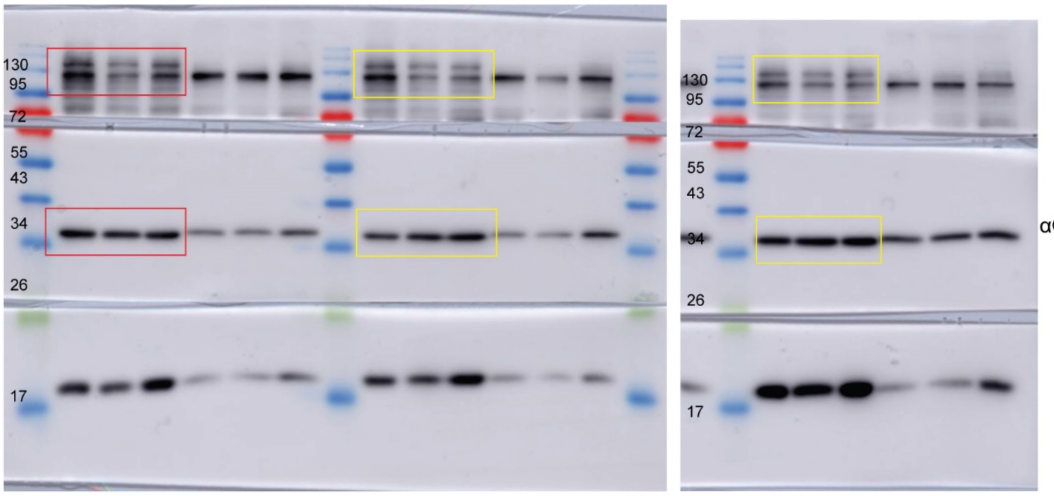

αGAPDH

αCofilin (not shown in this figure)

Figure S14A, experiment was performed on the same blot as Figure S8G

αFMNL2/3 (not shown in this figure)

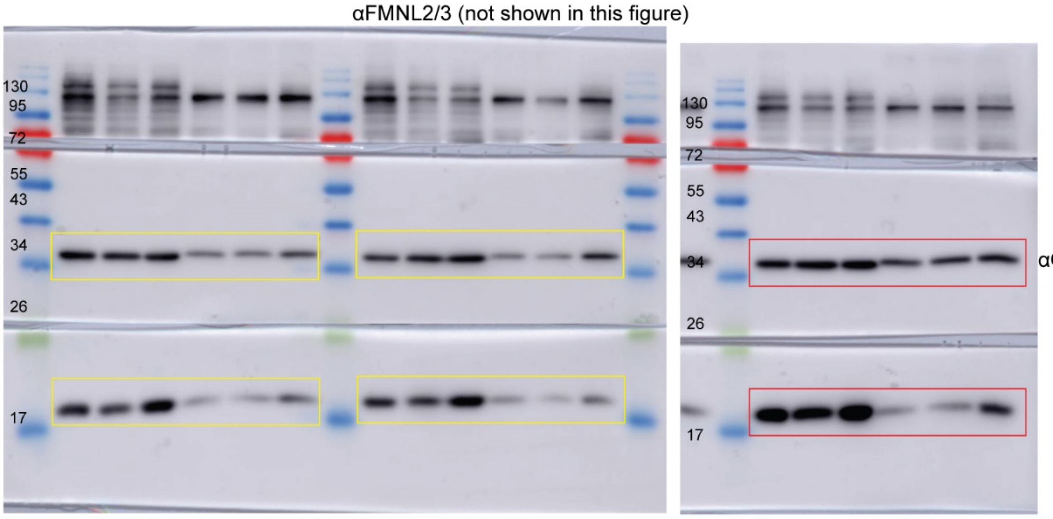

αGAPDH

αCofilin

Figure S14B, experiment was performed on the same blot as Figure S6D

αVinculin

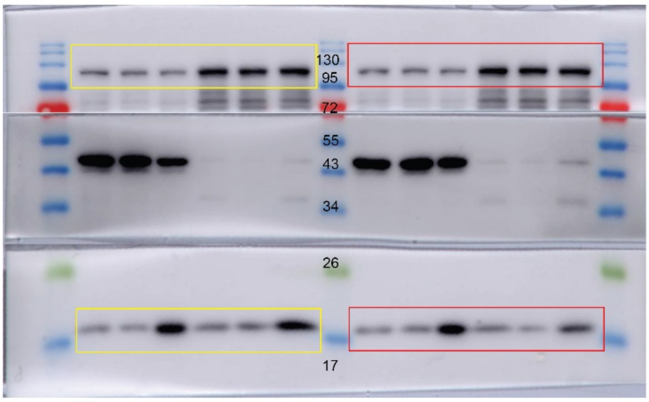

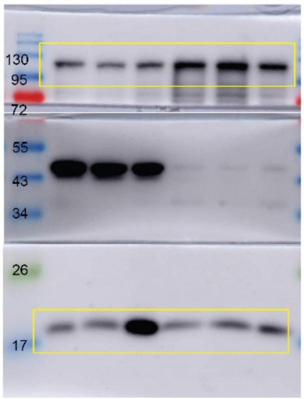

αArpC1b (only used for Figure S6D)

αp-Cofilin

Figure S15O

αVinculin αMena

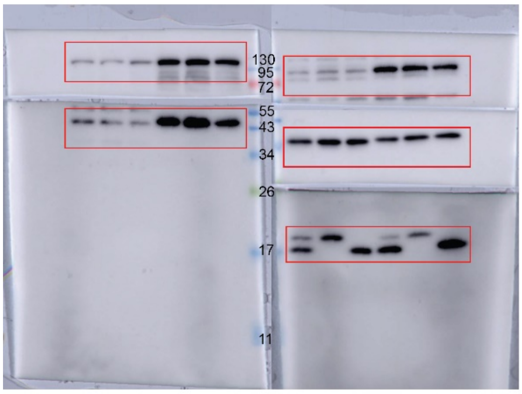

αGAPDH

αVASP αArpC5/L

αVinculin αMena

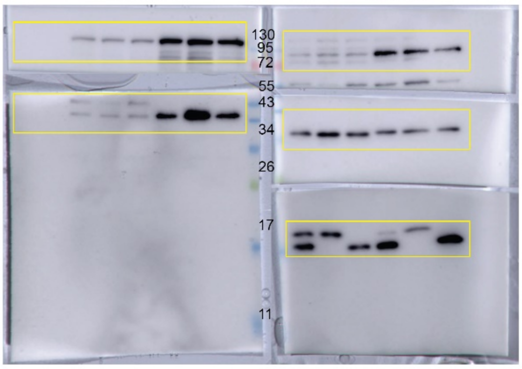

αGAPDH

αVASP αArpC5/L

αVinculin αMena

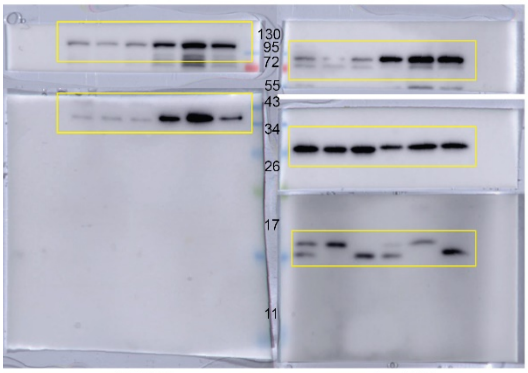

αGAPDH

αVASP αArpC5/L

Figure S16B

αVinculin

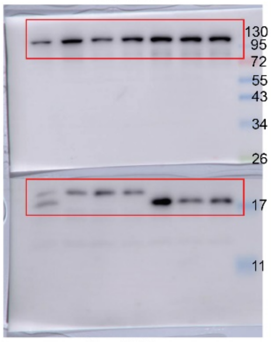

αArpC5/L

Figure S18: Uncropped Western blots

Chemiluminescence signals and color images of the PVDF membranes are overlaid. Corresponding figures, employed antibodies, and identity of the marker bands are annotated next to and on the blots, respectively. Red rectangles highlight areas shown in the corresponding figures. Yellow rectangles indicate repetitions used for statistical quantification of Western blots.

	Relative Protein Levels Compared to Respective Control											
Cell Line	ArpC5	ArpC5L	Arp3	Arp2	ArpC1b	ArpC1a	FMNL2	FMNL3	Cofilin	p-Cofilin	Mena	VASP
B16-F1 WT	1.00	1.00	1.00	1.00	1.00	1.00	1.00	1.00	1.00	1.00	1.00	1.00
B16-F1 C5KO #17		2.34±0.61↑↑	0.95±0.04	0.81±0.08	1.32±0.12↑	1.08±0.17	0.66±0.11↓	0.61±0.11↓	0.87±0.09	1.43±0.16↑	1.16±0.47	1.42±0.48↑
B16-F1 C5LKO #16	1.04±0.29		1.13±0.08	0.81±0.09	0.83±0.07	1.79±0.26↑	0.70±0.08↓	0.65±0.09↓	1.39±0.14↑	4.61±0.11↑↑	1.53±0.40↑	0.88±0.32
Rat2 WT	1.0	1.0	1.0	1.0	1.0	1.0	-	-	1.0	1.0	1.0	1.0
Rat2 C5KO #4	-	2.16±0.16↑↑	0.92±0.12	0.93±0.03	0.58±0.19↓	0.78±0.0.12	-	-	1.1±0.43	1.15±0.03	1.0±0.20	1.54±0.14↑
Rat2 C5LKO #11	1.93±0.32↑	-	1.78±0.25↑	1.25±0.13	1.10±0.50	1.38±0.20↑	-	-	1.45±0.47↑	2.25±0.28↑↑	1.11±0.40	0.71±0.12

Table S1: Quantification of Western blots

Average band intensities of 3 independent biological replicates after background subtraction, normalization via loading control and displayed as fraction/multiple of the respective WT control. Standard Errors of the Mean (SEM) are provided for all non-control groups. Reduction of expression below 0.7 times of the respective control is highlighted by ↓. Increase of expression above 1.3 times and 2 times are highlighted by ↑ and ↑↑, respectively.

Table S2: Full statistics for all figures

Descriptive statistics, normality tests, and test statistics for all quantification of cellular traits. Provided in separate file.

Movie S1: Comparison of B16-F1 wildtype, C5KO, and C5LKO branch junction structures

Movie S2: FRAP analysis of representative B16-F1 wildtype cell

Movie S3: FRAP analysis of representative B16-F1 C5KO #17cell

Movie S4: FRAP analysis of representative B16-F1 C5LKO #16 cell
